# Neuronal Nrf/SKN-1B controls a sexually dimorphic neuroendocrine pathway for *C. elegans* exploratory behaviour

**DOI:** 10.1371/journal.pgen.1012239

**Published:** 2026-08-13

**Authors:** Noviann McLean, Minaxi S. Gami, Nathan Dennis, Yasir Malik, Justin De Araujo, James Evans, Lydia Bennett, Marina Ezcurra, Jennifer M. A. Tullet

**Affiliations:** School of Natural Sciences, University of Kent, Canterbury, Kent, United Kingdom; Technion Israel Institute of Technology, ISRAEL

## Abstract

The SKN-1/Nrf transcription factor family is integral to metabolic homeostasis. There are three SKN-1 isoforms in *Caenorhabditis elegans*, each with different functions. One of these, SKN-1B, is expressed specifically in neurons and controls hermaphrodite behaviour in response to food. In a laboratory environment *C. elegans* hermaphrodites must allocate energy efficiently between exploratory food-seeking behaviour, reproduction, and survival, whereas males have to balance both food-seeking with mate-seeking exploratory behaviours. Here, we evaluate the role of SKN-1B in male behaviour and show that SKN-1B acts in a sexually dimorphic manner to control exploratory behaviour, mitochondrial morphology, and mitochondrial function. Moreover, we show that SKN-1B is expressed in a sexually dimorphic pattern, and that SKN-1B influences gene expression on a much broader scale in males compared to hermaphrodites. This analysis identified a neuronal signalling pathway, specifically in males between SKN-1B, the TGF-β ligand DAF-7 and the chemosensory regulator ODR-10, and that this pathway contributes to increased exploratory behaviour in *skn-1b* mutant males. Taken together our findings identify SKN-1B as a sex-specific neuronal switch to control food searching and mating behaviours. Sexual dimorphism is essential for optimizing survival and reproductive success and our findings establish SKN-1B as a regulator of sex-specific behavioural and metabolic traits. This work provides new insights into neuronal regulation of metabolism and behaviour, with implications for sex-specific physiological adaptations in other species.

## Author summary

All animals must make decisions about how to use their energy. They need to find food, reproduce, and stay healthy, but they cannot maximize all of these activities at the same time. Because males and females often have different reproductive priorities, they may use different biological mechanisms to balance these competing demands. Understanding how the brain coordinates these decisions is an important question in biology. We studied a gene called *skn-1* in the tiny roundworm *Caenorhabditis elegans*, a widely used model organism. One version of this gene, called *skn-1b*, is active in a small number of sensory neurons that help worms respond to their environment. We found that *skn-1b* affects males and hermaphrodites differently. In males, loss of *skn-1b* changes food-searching behaviour, alters the shape and performance of mitochondria and has widespread effects on gene activity. We also identified a signalling pathway that links *skn-1b* to genes involved in sensing environmental cues and controlling behaviour. Our findings show that *skn-1b* acts as a sex-specific regulator that helps coordinate behaviour and energy use. More broadly, this work reveals how the nervous system can tailor metabolism and behaviour to the different needs of each sex.

## Introduction

**Fig 1:**
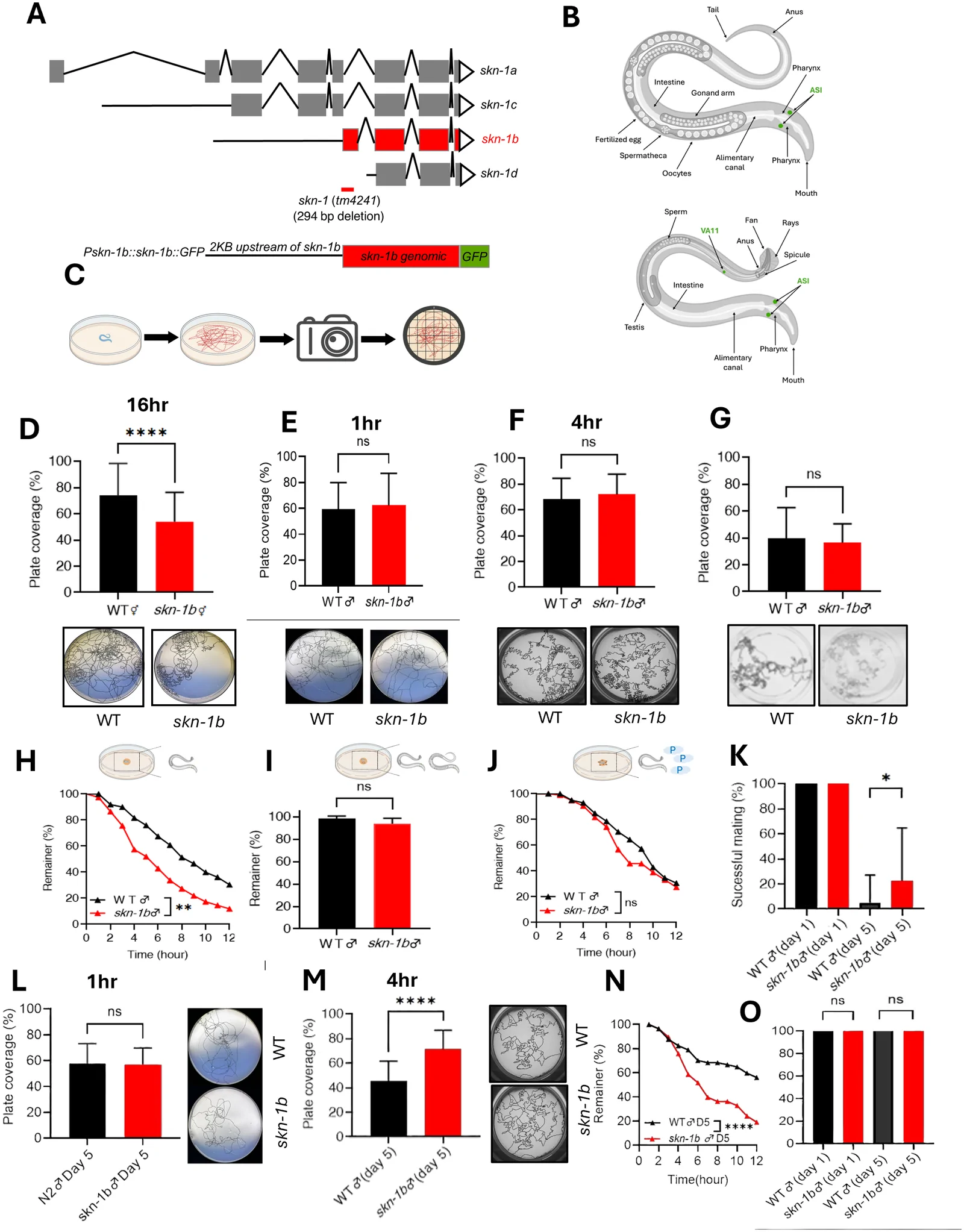
SKN-1B controls male exploratory and late-life mating behaviour. **A)** Representative images of an adult hermaphrodite and male *C. elegans* showing anatomical differences. **B)** *skn-1* genetic locus showing isoforms, *skn-1b(tm4241)* allele (294 bp deletion) and the SKN-1B::GFP reporter. *tm4241* reduces *skn-1b* mRNA specifically [4]. **C)** Exploration assay. Plates with single hermaphrodite animals are incubated for 16hrs; Males are incubated for 1 or 4 hrs (see methods and S1 Fig). **D-G)** Exploratory behaviour in WT and *skn-1b* mutants. Representative images of plates shown. **H-J)** Male leaving assays: **H)** Proportion remaining when individual males are placed on a spot of OP50 **I)** Proportion remaining when individual males are placed with single hermaphrodite **J)** Proportion remaining when individual males are placed on food spiked with hermaphrodite pheromone. **K)** Mating efficiency. **L-M**) Exploratory behaviour in day 5 males. **N)** Male leaving assay in day 5 males. **O)** Thrashing in liquid. Error bars indicate SD. ns, non-significant, * < 0.05, ** < p = 0.005, ****p < 0.05, Student’s t-test (**D-G, I, K-M, and O**). Log-Rank Mantel Cox test (**H, J and N**). Images generated in Biorender agreement number UN29WARSYX, http://BioRender.com/o0ejms4.

In mammals, Nuclear factor erythroid 2-related factors (Nrf) are crucial transcription factors that control antioxidant responses, detoxification and metabolism [1]. *C*. *elegans*, has only one sequence and functional Nrf orthologue, SKN-1, but its outputs are thought likely to be distributed between all the mammalian Nrfs [2]. *skn-1* is encoded on an operon with three isoforms (SKN-1A-C) confirmed *in vivo* (Fig 1A) [2]. SKN-1A and SKN-1C are expressed in the intestine and regulated, similarly to the Nrfs, at the level of cellular localisation [2]. In contrast, SKN-1B is expressed in the nuclei of two chemosensory neurons, the ASIs, which conduct some of the same functions as the mammalian hypothalamus including food sensing and metabolic control [3,4]. Recently, we identified a new role for SKN-1B in *C. elegans* exploratory behaviour [4]. SKN-1B acts by controlling a neuronal-physiological-behavioural axis, cell non-autonomously harnessing the endocrine TGF-β and insulin signalling pathways as well promoting a stable mitochondrial network organisation. These SKN-1B-mediated signalling and physiological responses act to promote food-seeking behaviour [4].

In the nematode *Caenorhabditis elegans*, individuals exist as either self-fertilizing hermaphrodites or males, each with distinct biological imperatives (Fig 1B) [5]. The self-fertilizing nature of hermaphrodites means that they must efficiently allocate energy between food intake, reproduction, and overall survival to maintain an optimal balance between energy storage and reproductive output [6]. By comparison, males, which rely on locating hermaphrodites for mating opportunities, must devote energy to exploration and mate-seeking behaviours while ensuring sufficient nutrient intake to sustain prolonged searching and copulation [6]. This balance between foraging, mating, and energy metabolism underscores the distinct physiological differences and behavioural strategies of each sex in *C. elegans* [7]. The efficiency and success associated with these behaviours is controlled by sex-specific neurological, endocrine, metabolic, and transcriptional mechanisms [8–12]. In particular, the Transforming Growth Factor-β (TGF-β) and Insulin endocrine signalling pathways communicate signals from between neurons and to somatic tissues to control behaviour [13]. Given the role of SKN-1B in hermaphrodite behaviours and endocrine signalling, we set out to test whether it had similar roles in males.

We demonstrate that neuronal SKN-1B acts in a sexually dimorphic manner to control *C. elegans* behaviour. We show that SKN-1B regulates food-driven exploratory activity sex-specifically, enhancing food-seeking in hermaphrodites while modulating mate-seeking behaviours in males. Notably, compared to WT controls, *skn-1b* mutant males maintain exploratory and mating behaviours into later adulthood, suggesting a role for SKN-1B in the regulation of age-related decline in male mating. Functional assays show that this sex-specific role of SKN-1B is also reflected in readouts of mitochondrial networks and mitochondrial function as well as by *skn-1b*-dependent changes in body wide mRNAs. Furthermore, we identify that increased levels of the TGF-β ligand DAF-7 in *skn-1b* mutant males leads to reduced expression of *odr-10*, a gene encoding a olfactory chemosensory receptor and established plasticity regulator of male feeding and mate searching behaviours [14] and that this neuronal signalling pathway contributes to the increased exploratory behaviour in *skn-1b* mutant males. Taken together our findings establish SKN-1B as a critical regulator of sexually dimorphic behavioural and physiological traits in *C. elegans* and identifies a SKN-1B mediated sex-specific neuronal switch to control food-seeking and mate-seeking behaviours. The neuronal role of mammalian Nrfs are not well characterised but this work implies that they may play roles in shaping sex-specific organismal physiology, metabolic and aging-related processes.

## Results

### SKN-1B controls appetite behaviour sex-specifically and promotes late-life mating in males

*C. elegans* can exist as either hermaphrodites or males (Fig 1B), with each sex facing unique biological challenges [5,6]. In relation to foraging, hermaphrodite *C. elegans* alternate between three behavioural states: food searching, food consumption, and satiety-induced quiescence [13]. These behaviours can be quantified by placing a single worm on a food plate and measuring the area explored (Fig 1C) [15]. The role of SKN-1B can be studied and distinguished from other SKN-1 isoforms using a *C. elegans* deletion mutant, that specifically blocks expression of the *skn-1b* isoform (Fig 1B) [4]. Previously, we found that animals lacking *skn-1b* exhibit reduced exploration compared to WT (Fig 1D) [12]. Males exhibit similar behaviours in relation to food searching but, in early adulthood, tend to explore more voraciously as they are also searching for a mate [6]. To test whether the behavioural role of SKN-1B was also maintained in male animals we compared WT and *skn-1b* mutant males in an exploration assay. Strikingly, compared to hermaphrodites, we found that both WT and *skn-1b* mutant males explored similarly, with a trend towards increased exploration in the mutants (Figs 1E-1F, S1). Moreover, this effect was maintained in the absence of food (Fig 1G). This suggests that SKN-1B acts sex-specifically to control exploratory, food-searching behaviour.

For males, exploration in the complete presence or absence of food could reflect either their food-seeking or mate-seeking behaviour and we hypothesised that SKN-1B may be implicated in determining the balance between these behaviours. To investigate this, we measured the time it took for WT and *skn-1b* mutant males to leave a small spot of food in search of a mate and found that *skn-1b* mutant males left the food spot faster than WT (Fig 1H). However, when provided with both food and a hermaphrodite, or food spiked with hermaphrodite pheromones, *skn-1b* mutant males remained on the food (Fig 1I-1J). Together, these data suggest that *skn-1b* mutant males have increased mate-searching behaviour and can sense both the physical and chemical presence of a mate.

While the hermaphrodite reproductive period is limited by self-sperm depletion and restricted to approximately 3 days, male *C. elegans* can identify and effectively mate with hermaphrodites throughout adulthood, although male mating does decline after 7 days of adulthood due to increased muscle excitability and a reduced ability to chemotax toward female pheromone [16]. We wondered whether the improved mate searching in *skn-1b* mutants could provide an advantage in old age. To determine the impact of *skn-1b* mutation throughout this period we measured the mating efficiency of WTs compared to *skn-1b* mutant males on both day 1 and day 5 of adulthood. We found that although mating efficiency was drastically reduced in the older groups, a higher percentage of *skn-1b* mutants retained their ability to mate at day 5 compared to WT controls (5% WT vs 22.5% *skn-1b* at day 5 p = 0.02 Fig 1K). We then tested whether the exploratory behaviour of *C. elegans* altered with age. We found that between days 1 and 5 of adulthood, WT male *C. elegans* exploratory behaviour decreased by ~30% (Fig 1E-1F and 1L-1M) indicating that their ability to search for a mate declines with age, correlating with a reduction in mating efficiency. However, *skn-1b* mutant males’ exploration did not decrease with age (Fig 1E-1F and 1L-1M). Furthermore day-5 *skn-1b* mutants also retained their enhanced food-leaving behaviour (Fig 1N). This suggests that *skn-1b* mutation confers an advantage for male mate-searching behaviour in old age.

Mating behaviour and success could have a causal link to the morphology of the male tail or sperm count. To test this, we compared the tail structures and numbers of spermatocytes in day 5 male WT and *skn-1b* animals using light and fluorescent microscopy respectively. However, we found no changes in overall tail morphology (rays, hook, spicules, cloaca) or in total numbers of sperm (S2A-S2B Fig). Taken together, these data show that SKN-1B is acting to suppress male mate searching and fertility under normal circumstances and suggests that this is due to an improved exploratory phenotype rather than due to alterations in tail morphology or germ cell number. Finally we explored the possibility is that *skn-1b* mutants are more active than WT animals, or display increased muscle excitability [17]. However we found no differences in liquid thrashing activity on either day 1 or day 5 compared to WT (Fig 1O). This is consistent with our previous finding in *skn-1b* mutant hermaphrodites and suggests that the foraging and mating behaviours observed in male *skn-1b* mutants derive from neuronal signalling rather than a muscle phenotype [4].

### Neuronal SKN-1B acts sex-specifically to control mitochondrial networks and function

**Fig 2:**
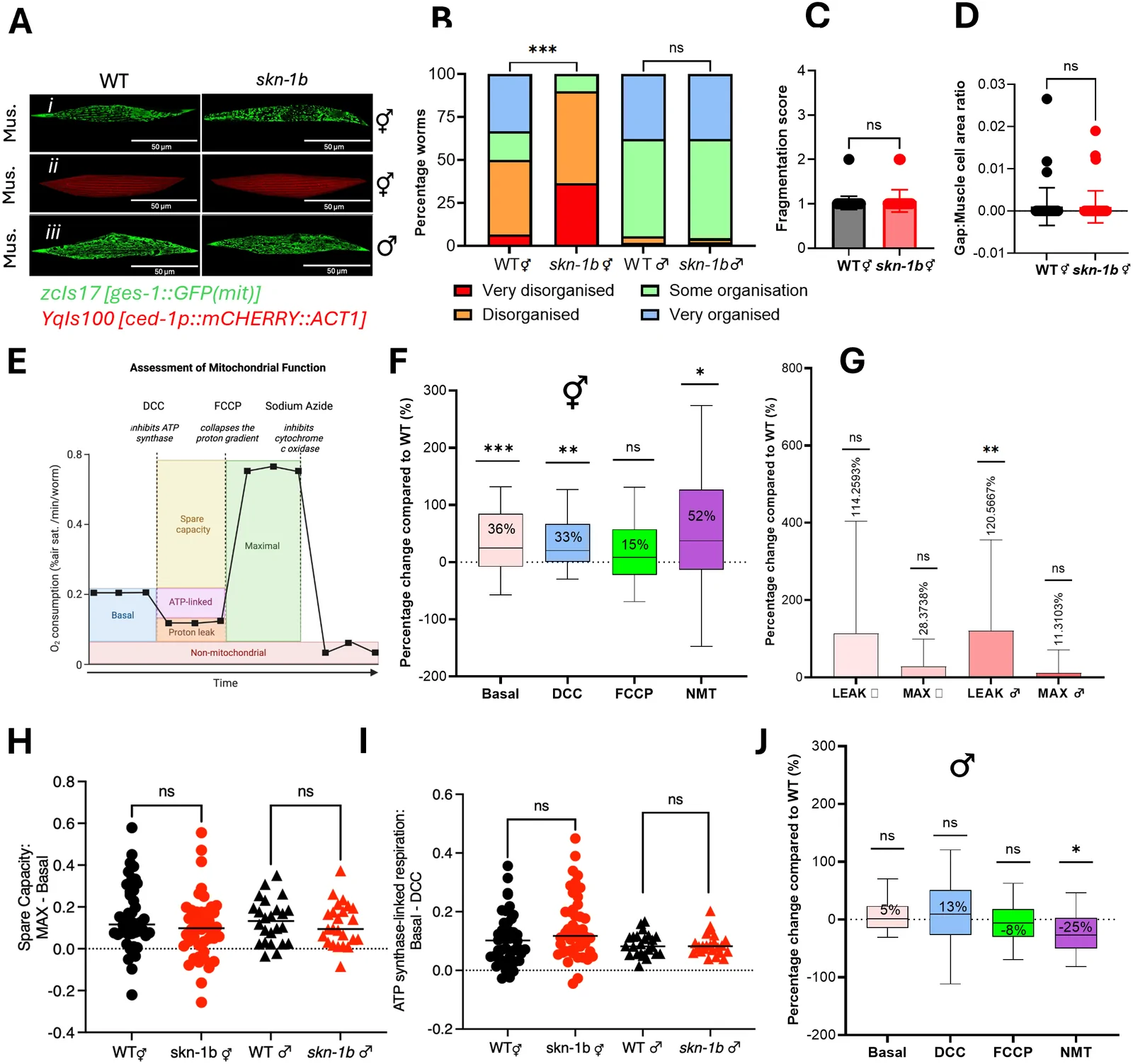
SKN-1B controls mitochondrial morphology and activity in a sex-specific manner. **A)** *i*) and ***iii*)** Mitochondrial morphology and *ii*) actin structures in WT and *skn-1b* mutants. Quantification of actin in S2B Fig) Qualitative analysis of hermaphrodite and male muscle mitochondrial networks. Scoring system shown in S2A. **C)** Qualitative scoring system of body wall muscle organisation in WT and *skn-1b* mutant hermaphrodites [58]. **D)** Quantification of the gap to total muscle cell area ratio between WT and *skn-1b* mutant hermaphrodites [59]. **E)** A representative O_2_ consumption curve generated from a respirometry experiment using PreSens OxoPlates [19]. Basal O_2_ consumption (OCR) is measured in normal conditions and in response to mitochondrial inhibitors. These values are used to calculate parameters of respiratory function: ATP synthase-linked respiration, proton leak, maximal (i.e., uncoupled) respiration and the mitochondrial spare capacity, and non-mitochondrial respiration. ATP-linked respiration = basal OCR – OCR after ATP synthase inhibition (DCC); proton leak = OCR after ATP synthase inhibition - non-mitochondrial OCR; maximal respiration = OCR after FCCP uncoupling; spare respiratory capacity = maximal – basal OCR; non-mitochondrial OCR = OCR after complete ETC inhibition (sodium azide). **F-J)** Mitochondrial O_2_ consumption in hermaphrodites and males. n=>30 animals per group **(B-J)**. Statistical comparisons were made using two-tailed t-test (**B, F-J**), or Chi-square test **(B-C)**. ns = non-significant, *p = 0.05, **p = 0.001, ***p = 0.0001. Error bars indicate SD**.**

Our previous work showed that neuronal SKN-1B acts in hermaphrodite muscle to promote organised mitochondrial networks in early adulthood, and that these changes directly influence *skn-1b*-mediated exploratory behaviour (Figs 2A-2B and S3A) [4]. Mitochondria act as metabolic centres within cells. They are dynamic organelles that change their network morphology, balancing their fission with fusion to maximise energy production [18]. Given the sexual dimorphism we observe with *skn-1b*-mediated exploration (Fig 1D-1F), we examined the mitochondrial structures in WT and *skn-1b* mutant male muscle using the *myo-3::GFP*_*mit*_ reporter to see if this could explain the phenotypes. Strikingly, and in contrast to the hermaphrodites, we found that both WT and *skn-1b* mutant male muscle mitochondria were very well organised (Figs 2A-2B and S3A). In muscle, mitochondria tend to align with muscle fibres. To assess whether the *skn-1b*-dependent mitochondrial changes in the hermaphrodites were due to altered muscle fibre structure, we examined a muscle-specific mCherry-tagged actin reporter in both WT and *skn-1b* mutant animals. However, there were no differences in organisation of actin fibres between WT and *skn-1b* mutants suggesting that SKN-1B affects muscle mitochondria directly (Figs 2A, 2C-2D and S3B-S3C). This suggests that *skn-1b* is acting to control muscle mitochondrial networks in a sexually dimorphic manner.

To determine whether these morphological changes impact mitochondrial function in either sex, we measured respiratory activity using PreSens OxoPlates as described in [19] under normal condition and in the presence of electron transport chain inhibitors (Fig 2E) [19]. Under normal conditions, hermaphrodite *skn-1b* mutants exhibited significantly elevated OCR (36% increase compared to WT, p=<0.001), indicating increased respiratory activity (Fig 2F). To assess ATP-linked respiration, we inhibited ATP synthase using N,N-Dicyclohexylcarbodiimide (DCC). Following DCC treatment, *skn-1b* mutant hermaphrodites also showed a 33% increase in OCR relative to WT (Fig 2F).

Sequential treatment with carbonyl cyanide-p-trifluoromethoxyphenylhydrazone (FCCP) and sodium azide was used to assess maximal and non-mitochondrial respiration, respectively. The non-mitochondrial OCR was significantly increased in *skn-1b* mutant hermaphrodites (52% increase vs WT; Fig 2F), indicating elevated background oxygen consumption. Despite these increases, *skn-1b* mutant hermaphrodites displayed no significant differences in proton leak, ATP-linked respiration, maximal respiration, or spare respiratory capacity compared to WT (Fig 2G–2I). Together, these findings indicate that although basal respiration is elevated, the overall capacity of mitochondria to respond to energetic stress is preserved [20].

Consistent with this, whole-body and muscle-specific ATP levels were unchanged in *skn-1b* mutants relative to WT (S4A-S4C Fig). The combination of increased oxygen consumption with unchanged ATP levels suggests reduced coupling efficiency, such that mitochondria require increased respiration to maintain normal ATP production. This is consistent with the observed mitochondrial morphological defects and indicates functional inefficiency rather than a loss of respiratory capacity.

In contrast, *skn-1b* mutant males showed no significant differences in basal OCR or in response to ATP synthase inhibition compared to wild type (Fig 2J), indicating that respiratory activity is largely unaltered under these conditions. However, the non-mitochondrial OCR was significantly reduced in mutant males (25% decrease vs WT; p < 0.05; Fig 2J), suggesting a lower level of background oxygen consumption and a potentially improved bioenergetic profile [20]. Further analysis revealed that *skn-1b* mutant males exhibited a marked increase in proton leak (~120% increase vs WT), while maximal respiration, spare respiratory capacity, and ATP-linked respiration remained unchanged (Fig 2G–2I). Although increased proton leak typically reflects reduced coupling efficiency, the absence of changes in ATP levels (S4C-S4D Fig) and other respiratory parameters suggests that overall mitochondrial function is maintained [20–22].

Taken together, these data reveal a clear sex-specific role for SKN-1B in mitochondrial regulation. In hermaphrodites, loss of SKN-1B leads to increased basal respiration and reduced coupling efficiency, consistent with functionally compromised mitochondria despite preserved respiratory capacity. In contrast, males exhibit largely intact mitochondrial function, with only selective changes in proton leak and non-mitochondrial respiration. These findings suggest that SKN-1B is required to maintain efficient mitochondrial function specifically in hermaphrodite muscle, but is largely dispensable in males.

### Neuronal SKN-1B acts to control sexually dimorphic body-wide transcription

Given the strong sexual dimorphism in *skn-1b*-mediated phenotypes we hypothesized that loss of neuronal *skn-1b* would drive different gene expression profiles in hermaphrodites and males, reflecting the known sexually dimorphic behaviours and neuronal functions in *C. elegans* [23,24]. In hermaphrodites, SKN-1B is expressed in the chemosensory ASI neurons, and we observed identical SKN-1B::GFP expression in male ASIs (S5A-S5B Fig). Interestingly however, in males, SKN-1B::GFP expression also appeared in an additional cell near the tail fan (S5C Fig). Co-expression of SKN-1B::GFP and the NeuroPAL reporter identified the additional male tail cell as the VA11 neuron (S5C Fig) [25]. This sexually dimorphic expression pattern implies that VA11 may contribute to SKN-1B-mediated male mating behaviour [26,27].

**Fig 3:**
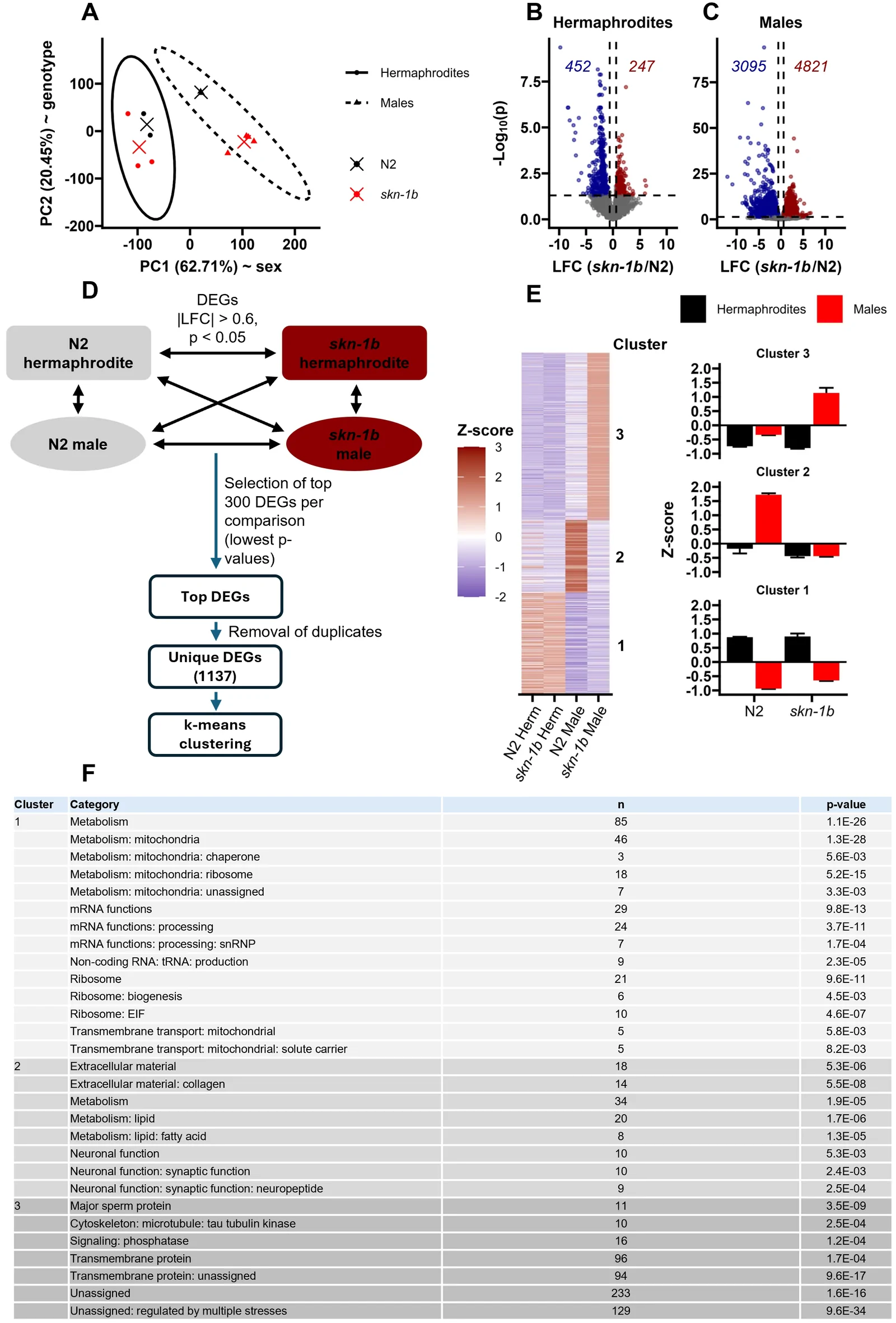
SKN-1B controls sex-specific transcriptional programmes. **A)** Principal components analysis (PCA) of RNA-Seq data showing *skn-1b* (red), wild-type (N2; black), hermaphrodite (circles) and male (triangles) samples. The ellipses were fitted to the male (dashed) and hermaphrodite (solid) data, respectively, and represent the 95% confidence intervals. Crosses denote the group centroids (mean PC scores on both axes). **B-C)** Volcano plots showing *skn-1b*-dependent genes in hermaphrodites (B) and males **(C)**. The vertical and horizontal lines represent the LFC (<-0.6 or >0.6) and FDR-adjusted p-value (<0.05) cutoffs, respectively. Gene counts of up- and down-regulated genes are provided. Validation of gene expression changes is in S6 Fig. **D)** Workflow for the extraction of relevant differentially expressed genes (DEGs) for k-means clustering. **E)** Heatmap and average z-scores of k-means clusters. Data are presented as mean (heatmap) and mean + /- standard error (bar chart). **F)** Gene set enrichment analysis of k-means clusters using WormCat. Only terms with Bonferroni-adjusted p-values <0.05 are shown.

Despite this limited expression, *skn-1b*-phenotypes suggest effects in multiple tissues. We therefore examined the transcriptional activities of day-1adult WT and *skn-1b* mutant hermaphrodites and males using RNA Sequencing [4]. We found that the transcriptomes of hermaphrodite, male, WT and *skn-1b* mutant samples clustered independently by principal components analysis (PCA; Fig 3A), with PC1 primarily separating hermaphrodite and male samples, and PC2 primarily separating WT and *skn-1b* samples. However, inter-genotype distances on both PC1 and PC2 were far greater between male samples (mean distance PC1 = 82; PC2 = 105) than hermaphrodite samples (mean distance PC1 = 15; PC2 = 48) indicating that *skn-1b* has pronounced sexually dimorphic effects on the *C. elegans* transcriptome. Supporting this, differential expression analysis identified just 699 *skn-1b*-dependent differentially expressed genes (DEGs; log_2_ fold-change < -0.6 or > 0.6, p < 0.05) in hermaphrodites compared to 7916 in males (Fig 3B-3C, S1 Table, S6A-S6B Fig). In contrast, the effects of sex only differed slightly in magnitude between WT and *skn-1b* samples, with 8035 DEGs in WT animals and 11475 in *skn-1b* mutants (S7A-S7B Fig, S1 Table). Additionally, a like-for-like comparison of our WT male-enriched genes with a published male-enriched gene set revealed a substantial and highly significant overlap with a similar number of DEGs (S7C Fig).

To gather a comprehensive picture of the effects of *skn-1b* on the transcriptomes of animals of both sexes, we performed k-means clustering with a subset of the most statistically significant DEGs derived from all possible between-group comparisons in our dataset (Fig 3D). A total of 1137 genes were selected, and clustering performed on the normalised and scaled read matrix (S2 Table), and the optimal number of clusters was determined using the Silhouette Method [28] (S8A-S8B Fig). Three clusters were identified which best captured the transcriptional variation in the gene set (Fig 3E): a 339-gene cluster with higher expression in hermaphrodites of both genotypes (cluster 1), and two clusters with diverging expression patterns in WT and *skn-1b* males (clusters 2 and 3, with 241 and 557 genes, respectively). Cluster 1 also showed slight differences in activity between *skn-1b* and WT males, but no cluster clearly differentiated *skn-1b* and N2 samples across both sexes or in hermaphrodites alone, further indicating that the effects of *skn-1b* on the *C. elegans* transcriptome are highly sexually dimorphic and largely limited to males.

To determine the likely functions and tissue distributions of these co-regulated gene clusters, we performed gene set enrichment analysis (GSEA) and tissue enrichment analysis (TEA), using WormCat and the WormBase TEA tool, respectively [29]. This analysis indicated that all identified gene clusters diverged significantly in both function and distribution (Fig 3F; S3 Table). Genes with higher expression in hermaphrodites of both genotypes (cluster 1) were enriched for a variety of terms related to mRNA processing and translation, protein trafficking, mitochondrial proteostasis, mitochondrial solute import, and mitochondrial metabolism. These genes were overrepresented in the reproductive, muscular, intestinal and nervous systems, and the germline, and 158/339 genes were identified as hermaphrodite-enriched. In contrast, cluster 2, consisting of genes highly expressed in WT males, were enriched for terms related to lipid metabolism, neuropeptide-related neuronal functions, and collagens, and were overrepresented in the coelomic system, the preanal hypodermis (hyp12), and the AWC neurons. Lastly, cluster 3, consisting of genes with higher expression in males of both genotypes, with highest expression in *skn-1b* males, was enriched for terms related to unassigned stress responses, transmembrane proteins, cytoskeletal processes, and the major sperm protein. TEA indicated these genes were enriched in the reproductive and epithelial systems, the germline, and the amphid sheath cell, and 384/557 genes were identified as male-enriched. Overall, the over representation of hermaphrodite-enriched and male-enriched genes in clusters 1 and 3, respectively, reflect the fact that the expression of these genes is largely determined by sex. However, it is interesting that, in both cases, there are also differences in expression between *skn-1b* and WT males, particularly for cluster 3, suggesting that SKN-1B’s role in males is not limited to sex-specific gene expression but may also contribute more broadly to the regulation of male transcriptional programs.

Since our k-means clustering analysis failed to delineate the more subtle effects of *skn-1b* on the transcriptional activity of hermaphrodites, we also performed separate GSEA and TEA of *skn-1b*-regulated DEGs in hermaphrodites (S3 Table). GSEA of upregulated genes only uncovered one enriched term, “signalling”, but TEA indicated that they were overrepresented in several cells and tissue types, most notably the PVD and OLL neurons (with 93 and 94 genes bearing these terms, respectively), and the pseudocoelomic GLR cells (48 genes). Downregulated genes, in contrast, were enriched for extracellular material and collagens, unassigned transmembrane proteins, and unassigned stress responses, and were overrepresented in a variety of body systems, including the hypodermis, epithelial system, excretory system, the postdeirid sensillum, and the reproductive system.

This analysis did not identify any DEGs in the ASI neurons. However, comparison of our data set with genes identified as ASI enriched in CeNGEN (2055 in hermaphrodites and 1689 in males), 611 and 391 overlap with our sex-specific *skn-1b* DEGs respectively (S3 Table). Taken alongside our k-means clustering analysis, this indicates that SKN-1B regulates transcriptional activity in a wide range of body systems in both hermaphrodites and males, and could act either cell autonomously or non-autonomously.

Given the established role of other SKN-1 isoforms in controlling stress responses, and the indication that some SKN-1B targets are associated with this term, we checked whether the genes in our lists corresponded to known SKN-1 targets [30–32]. We found significant overlaps with these targets in both our hermaphrodite and male gene lists (S4 Table), though the majority of SKN-1B-regulated genes were unique to this study. We also checked whether SKN-1 mRNA was altered in *skn-1b* mutants and found it to be elevated in both males and hermaphrodites (both LFC > 0.96), though in neither case was this statistically significant (both p > 0.083). This indicates that while SKN-1B controls many of the same genes as other SKN-1 isoforms, it is likely to also have an independent function.

### SKN-1B acts to control mitochondrial and behavioural genes

The enrichment of several mitochondrial terms, in particular mitochondrial metabolism, in cluster 1 supports our mitochondrial function and morphology data (Fig 3E-3F). To further explore the role of SKN-1B in mitochondrial function, we identified *skn-1b*-dependent genes specifically implicated in mitochondrial function and morphology using a combination of Simple Mine and the STRING data base. In hermaphrodites we only identified 5 DEGs with mitochondrial roles, but in males there were 86 (S5 Table). This latter group included genes implicated in the Electron Transport Chain (ETC), tricarboxylic acid (TCA) cycle, mitochondrial morphology and metabolism. This included subtle upregulation of well characterized genes implicated in mitochondrial fission (*drp-1* L2FC 1.28) and fusion (*eat-3* L2FC 1.02) but only 9/86 mitochondrial genes were altered >2 L2FC. Thus, we suggest that the magnitude of these changes is unlikely to be sufficient to account for the functional mitochondrial changes in *skn-1b* mutant males, and given that *skn-1b* mutant males exhibit ‘normal’ looking mitochondrial structures, that this is the result of a broader compensatory response.

To understand the impact of SKN-1B in male mating behaviour, we mined CeNGEN for the precise neuronal expression pattern of *skn-1b*-dependent transcripts. This identified *skn-1b*-dependent changes in mRNAs across inter, motor and sensory neuron types, some of which are shared between hermaphrodites and males and some that are male specific (S6 Table). Indeed, removal of *skn-1b* causes changes in genes expressed in neurons that have been implicated in ascaroside pheromone detection (e.g., ADF, ASH, AWA), mate finding and searching (e.g., CEM, PHA, PHB), vulval location (e.g., HOB, PCA, PCC), sperm ejaculation (SPD, SPV) and other aspects of precise mating behavior (e.g., PHD, PVD, PVV, PVX, PVY) [12,33]. This included changes in the well-characterized genes *lov-1* and *pkd-2*; two polycystins associated with male chasing, vulval recognition and contact, in CEM, HOB and R8B neurons [34–36]. This suggests that SKN-1B function is embedded in a variety of male mating behaviors across several neuronal classes and sub classes.

**Fig 4:**
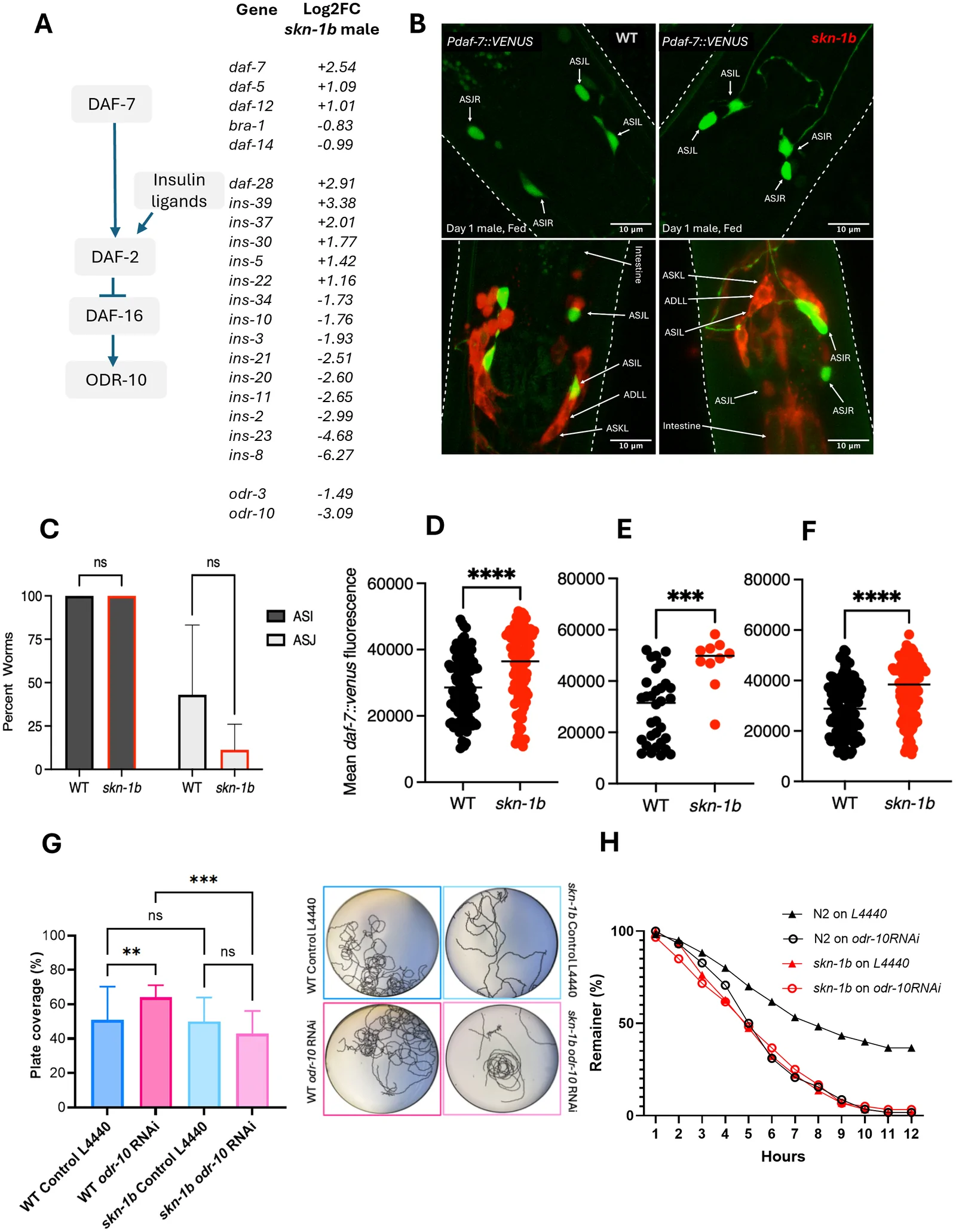
SKN-1B controls DAF-7 and *odr-10* levels to control male exploratory behaviour. **A)** Expression changes of TGF-β signalling, Insulin signalling and odorant associated mRNAs in *skn-1b* mutant males. **B-F)** DAF-7::Venus expression in ASI and ASJ neurons of WT and *skn-1b* mutants. Representative images show the DAF-7::Venus expression pattern, with DiI staining (red) showing amphid neurons **(B).** The percentage of animals observed with DAF-7::Venus expression in the ASI or ASJ neurons was recorded **(C),** and the levels of DAF-7::Venus in each neuronal population quantified separately **(D-E)** and together **(F). G-H)** Exploratory behaviour **and** male leaving assay in WT and *skn-1b* mutants with and without *odr-10* RNAi. Representative images of plates shown. **B-G)** ≥3 biological replicates, n=<30 worms. Statistical comparisons, two-tailed t-test ****P < 0.0001, ***P < 0.001 and two-way ANOVA, ns = non-significant; **H)** Log-Rank Mantel Cox test.

To gain further insight as to whether any *skn-1b*-dependent transcriptional changes could account for the sexually dimorphic differences in exploratory behaviour that we observed, we cross referenced our gene lists with those assigned terms linked to exploratory behaviour, e.g., ‘movement variants’ in the STRING database. In hermaphrodites there were 11 genes in this category but in the males, this provided a list of 656 genes, 500 which were up-regulated and 156 down-regulated in *skn-1b* mutants (S7 Table). These genes could be categorized further into those implicated in muscle or neuronal function, extracellular matrix or adhesion or signaling, although some genes crossed into more than one category (S8A-S8B Fig). Interestingly, this analysis showed *skn-1b*-dependent changes in the TGF-β and insulin signaling pathways (Fig 4A), both of which have previously been implicated in male mate searching behaviors [4]. Specifically, *skn-1b* mutant males showed altered expression of the gene encoding the TGF-β ligand *daf-7*, and several genes encoding insulin receptor ligands including *daf-28, ins-39 and ins-37* (Fig 4A) [37,38]*.* Additionally, the expression of *odr-10* a seven transmembrane domain food chemosensory receptor, that controls male feeding and mate searching behaviours, downstream of the TGF-β and insulin signalling was also down-regulated Log_2_ (fold Change) -3.09 p=<0.05) in *skn-1b* mutant males (Fig 4A) [14,39–41]. This suggests that neuronal SKN-1B is acting to control both the TGF-β and insulin neuroendocrine signaling pathways, and this, coupled with increased levels of genes implicated in male sex-drive co-ordinate to control exploratory and mating behavior in *C. elegans* males.

### SKN-1B regulates male-specific neuroendocrine signalling to control exploratory behaviour

**Fig 5:**
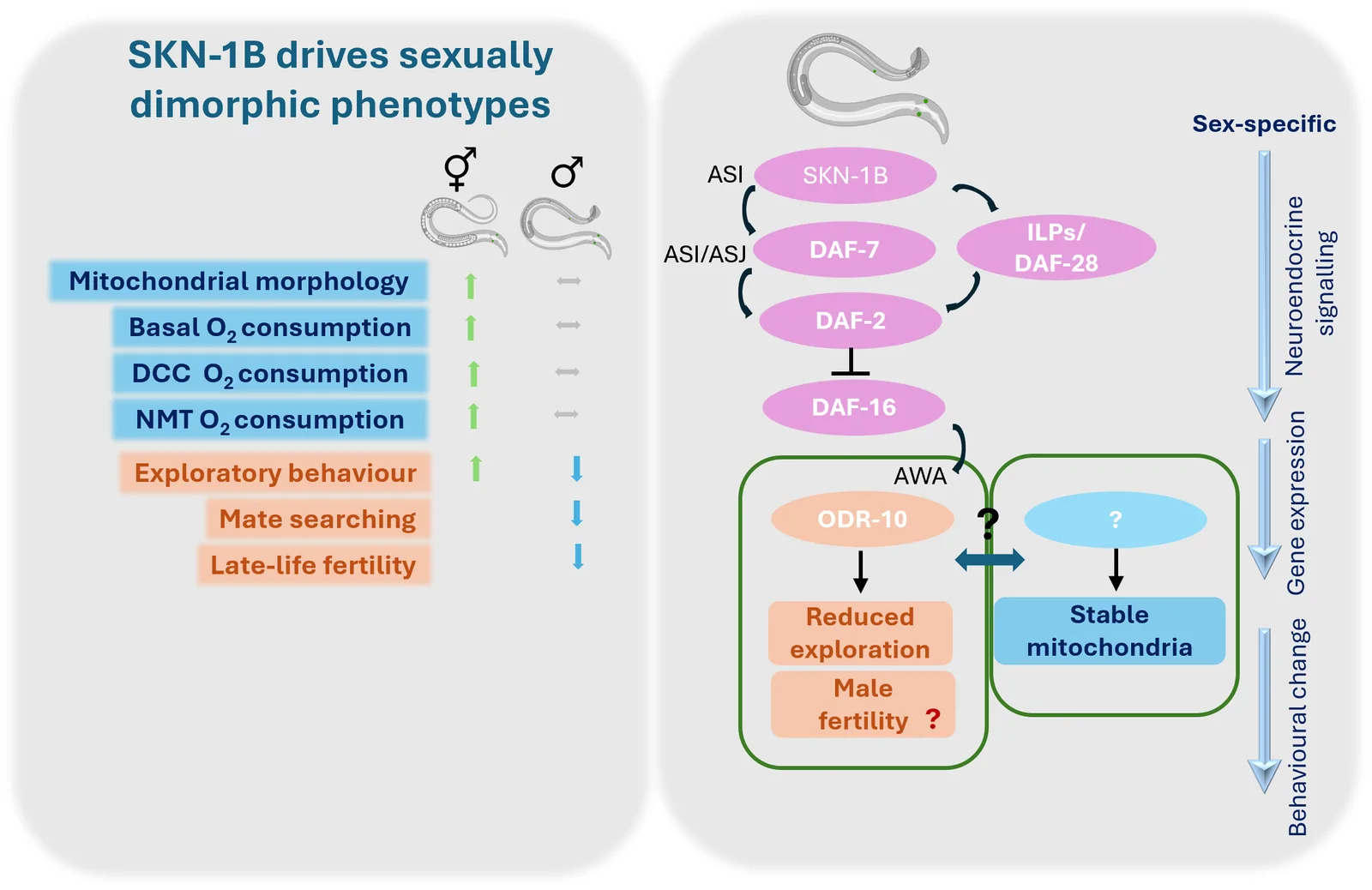
Neuronal SKN-1B acts as a sexually dimorphic molecular switch to balance hermaphrodite and male exploration and male mating behaviours throughout life. SKN-1B controls *C. elegans* bioenergetics and behaviour in a sexually dimorphic manner. SKN-1B-dependent gene expression profiles are sex-specific, although interactions with TGF-β and insulin signalling downstream of neuronal SKN-1B occur in both sexes [4]. The phenotypic associations of *skn-1b*-dependent genes, combined with our functional analysis strongly support a role for SKN-1B in sex-specific exploratory and mating behaviours driven by a *daf-7*, *odr-10* neuronal circuit. Images generated in Biorender agreement number UN29WARSYX http://BioRender.com/o0ejms4.

In males, ODR-10 is controlled by neuroendocrine signaling and high levels of the TGF-β ligand DAF-7 in the ASI and ASJ chemosensory neurons likely inhibits insulin signaling and DAF-16/FoXO activity, down-regulating *odr-10* expression [14]. This decreased expression of *odr-10* promotes mate-searching behavior [14,42]. Thus, we hypothesized that alterations to endocrine signaling and a subsequent downregulation of *odr-10* may contribute to the enhanced exploratory behavior of *skn-1b* males. To test the hypothesis, we quantified levels of the TGB-β ligand DAF-7 in *skn-1b* mutant males using confocal microscopy. As expected, we observed expression of a DAF-7::Venus reporter in both the ASI and ASJ neurons in WT males, although the percentage of animals with ASJ expression was less than those with ASI expression (Fig 5B-5C). Quantification of these images showed that DAF-7::Venus levels were increased in *skn-1b* mutant animals in both the ASI and ASJ neurons (Fig 5D-5E). As *odr-10* expression is thought to be influenced by the overall levels of DAF-7, we also quantified the combined neuronal expression levels of DAF-7::Venus and found these to be increased in *skn-1b* mutant males, corroborating the increase levels of *daf-7* mRNA in these animals (Fig 5A and 5F). These data, together with the ~ 3-fold decrease in *odr-10* mRNA observed in our data set, strongly implies that neuronal SKN-1B is required to mediate the neuroendocrine regulation of *odr-10* in males.

Finally, we tested whether this decrease in *odr-10* expression can explain the altered behavior observed in *skn-1b* males (Fig 1E). We tested the impact of *odr-10* RNAi knockdown on WT and *skn-1b* male exploratory behavior under fed conditions. WT males fed *odr-10* RNAi showed a modest but significant increase in their exploratory behaviour supporting the idea that decreasing *odr-10* levels promotes exploration (Fig 5G). Interestingly however, although both WT and *skn-1b* mutant males fed on control RNAi plates had the same level of exploratory behaviour, *skn-1b mutation* and *odr-10* RNAi were non-additive with respect to exploratory behaviour (Fig 5G). Moreover, similarly to *skn-1b* mutation *ord-10* RNAi caused increased food-leaving behaviour in males, but these effects were also non-additive (Fig 5H). Together with the expression data, these data imply that *skn-1b* and *odr-10* interact to control exploratory behaviour in males, and drive mate searching, and that this could account for the SKN-1B mediated sexually dimorphic effects on exploratory behaviour and possibly contribute to the improved late-life mating phenotype associated with *skn-1b* mutation.

## Discussion

*C. elegans* hermaphrodites and males elicit behavioural differences, including food searching, pheromone preference, complex cue decision-making and learning [6]. Our findings demonstrate that neuronal SKN-1B exerts sex-specific control over transcriptional programs, mitochondrial dynamics, and behaviour (Fig 5).

### The impact of SKN-1B/Nrf on whole-body gene transcription is greater in male *C. elegans*

Our data shows a striking, and surprising, difference in *skn-1b*-dependent transcriptional change between males and hermaphrodites. The limited transcriptional change in hermaphrodites suggests either that SKN-1B acts more subtly in modulating their metabolic processes, potentially prioritizing reproductive energy efficiency, or that SKN-1B controls protein level changes to mediate its phenotypes, which are not picked up using RNA Sequencing. In contrast, the dramatic changes in gene expression observed in *skn-1b* mutant males, suggests either that SKN-1B plays a more expansive role in male physiology or that loss of *skn-1b* in male elicits a larger compensatory response. Alternatively, it is possible that these changes in gene expression are mediated by male-specific expression of SKN-1B in the VA11 neuron. VA11 forms neuro-muscular junctions with the body wall muscles and is implicated in locomotion, specifically backwards movements [43]. Although not directly linked to male mating, it is connected to the post cloacal sensilla neurons (PDB, PDC or PVV) which are responsible for controlling movement related to copulation, and the hook sensillum neurons (HOA and HOB) which are important for vulva sensing – although ablation of this neuron did not cause any mating-related phenotypes [26,27]. Overall, the sex-specific neuronal expression and transcriptional effects of SKN-1B reinforce the notion that it coordinates behavioural responses cell autonomously and non-autonomously to optimize survival and reproductive success in each sex.

### SKN-1B/Nrf as a sexually dimorphic metabolic regulator

Our data shows that SKN-1B influences mitochondrial organization and function in a sex-specific manner. In hermaphrodites, SKN-1B appears to promote structured mitochondrial networks in muscle whereas, in males, SKN-1B has no impact on mitochondrial networks (Fig 2A-2B). Mitochondrial networks have been demonstrated to impact behaviour in *C. elegans*, and indeed genetic mutations that reverse disordered networks are sufficient to restore normal exploratory behaviour [4,44]. Thus, it is possible that male *C. elegans* overcompensate in the absence of *skn-1b* to ensure that mate searching behaviour is maintained. This would support a model whereby SKN-1B modulates mitochondrial function in a manner that aligns with the distinct reproductive and survival strategies of each sex. A recent study showed male neurons in *C. elegans* undergo major metabolic changes early in adulthood [45]. Our data shows that the transcriptional changes associated with metabolism in male *skn-1b* mutants is above and beyond even these changes. Thus, our data imply that the positive impact of *skn-1b* on late-life mating is due to a more dramatic and potentiated impact of these widespread metabolic changes in the mutant animals, indirectly promoting mating efficacy.

We also show that the altered mitochondrial networks in *skn-1b* mutant hermaphrodite muscles correlates with changes in mitochondrial function consistent with inefficient respiratory activity and hallmarks of a low bioenergetic health index (Fig 3E-3I) [20]. However, the essentially ‘normal’ state of *skn-1b* mutant male mitochondria suggests that sexual dimorphism is an important consideration when studying mitochondrial dynamics. Dysregulated mitochondrial structures or function is complex, and a naive interpretation would be that dysregulation is metabolically bad for an organism and in this scenario that *skn-1b* mutant hermaphrodites are at a disadvantage compare to males. However, modulating mitochondrial proton leak is a therapeutic target for complex metabolic diseases including obesity and diabetes in humans, with a higher proton leak correlating to a higher basal resting metabolic rate, energy expenditure, and weight loss [46]. Further studies are required to understand the physiological impact of these sex-specific effects.

It is counter intuitive that the changes in mitochondrial morphology and function are not mirrored by changes in mitochondrial gene expression in hermaphrodite animals. Instead, *skn-1b* mutant males, where mitochondrial phenotypes are essentially normal exhibit changes in mitochondrial-associated gene expression (S5 Table). The involvement of some of these in mitochondrial morphology or metabolism could suggest a neuronally driven programme of gene expression that compensates for SKN-1B driven effects in a mutant background.

Despite the sexually dimorphic phenotypes of *skn-1b* mutants, similar endocrine signalling pathways are stimulated in both sexes. Our previous work has showed that *skn-1b* mutant hermaphrodites have elevated levels of neuronal DAF-7 and interacts with both TGF-β and Insulin signalling to control behaviour [4]. In males SKN-1B controls both DAF-7 and insulin ligand expression, which in males converge on the insulin pathway to control DAF-16 cellular localisation and activity. Thus, one outcome of this is the downregulation of *odr-10* and increased movement of *skn-1b* mutants. *odr-10* levels are not altered in *skn-1b* mutant hermaphrodites, so sex-specific neuroendocrine signalling likely explains the SKN-1B-dependent impact on sexually dimorphic behaviours.

### Neuronal SKN-1B/Nrf and late-life reproductive health in males

We also show that SKN-1B influences sex-specific behaviours. Whilst in hermaphrodites it acts to enhance food-seeking behaviour, in males it has no impact. In contrast, the role of SKN-1B in male behaviour is linked to mate-seeking. The SKN-1A and SKN-1C isoforms have been widely associated with lifespan and age-related health [2,47]. In contrast, SKN-1B is not required for the normal lifespan of fully fed *C. elegans* but has been implicated in the increased lifespan incurred when *C. elegans* undergo certain dietary restriction regimes [3,4]. However, overall lifespan is not always indicative of age-related health and organisms undergo ageing in different tissues at different rates. Reproductive decline is one aspect of ageing, but our previous work did not identify any role for SKN-1B in hermaphrodite progeny production [4]. However, one of the surprising findings of this work has been that loss of *skn-1b* improves male age-related reproductive health. Male fertility is complex and relies on pheromone detection, chemotaxis, sperm health and mechanical interactions between the male tail and hermaphrodite vulva [6,35]. Our analysis identified that both *lov-1* and *pkd-2*, which encode two polycystins associated with male chasing, vulval recognition and contact, [34–36] as well as *tax-6* known to promote male mating behavior [48] are all upregulated in *skn-1b* mutant males (S1 Table). Therefore, the increased expression of these genes may contribute to the increased fertility of *skn-1b* mutant males.

Taken together, our findings establish neuronal SKN-1B as a crucial determinant of sexually dimorphic metabolic and behavioural regulation in *C. elegans*. By coordinating transcriptional programs that balance energy metabolism, foraging, and reproduction in a sex-specific manner, SKN-1B ensures physiological adaptation to the unique challenges faced by hermaphrodites and males (Fig 5). The sex-specific roles of other SKN-1 isoforms has not been explored, but given their broad functionality and our findings here, this may offer opportunities for further discovery. Moreover. the role of mammalian Nrfs in the brain is not well characterised but this work suggests a role for Nrf transcription factors in shaping sex-specific metabolic and behavioural traits across species, with potential implications for understanding the neuronal regulation of metabolism and aging in more complex organisms.

## Methods

### *C. elegans* husbandry, strains, and RNAi

The worms used for all assays were maintained at 20°C on nematode growth media (NGM) plates and seeded with *E. coli* OP50 [49]. For all experiments, strains were grown on *E.coli* OP50 for at least three generations without starvation. Strains used: wild-type N2 CGCM, GA1058 *skn-1b(tm4241)*,), LIU1 *ldrIs1[dhs-3p::dhs-3::GFP + unc-76(+)]*, JMT202 *skn-1b(tm4241) [dhs-3p::dhs-3::GFP + unc-76(+)]*, GA1017 *wuEx217[Pskn-1b::skn-1b::GFP; rol-6]*, NeuroPAL *otIs669 [25,50]*, *wuEx217[Pskn-1b::skn-1b::GFP; rol-6]*; *otIs669*, SJ4103 *zcIs14 [myo-3::GFP(mit)], JMT90 skn-1b(tm4241); zcIs14 [myo-3::GFP(mit)],* SJ4143 *zcIs17 [ges-1::GFP(mit)],* JMT203 *skn-1b(tm4241) zcIs17 [ges-1::GFP(mit)]*, NM4244 *jsIs609 [Pmec-7::GFP(mit)],* JMT216 *skn-1b(tm4241) jsIs609 [Pmec-7::GFP(mit)],* WX8490 *YqIs100 [ced-1p::mCHERRY::ACT1],* JMT204 *skn-1b(tm4241) YqIs100 [ced-1p::mCHERRY::ACT1], GA2001 wuIs305 [myo-3p::Queen-2m],* PHX10467 *skn-1b(syb10467); wuIs305 [myo-3p::Queen-2m]; JMT50 drcSI7[unc-119;Pdaf-7::Venus], JMT75 skn-1b(tm4241) drcSI7[unc-119;Pdaf-7::Venus].* All WT *vs skn-1b* mutant experiments compared N2 CGCM and *skn-1b(tm4241)*. Males were isolated from the CGCM strain and maintained by crossing with CGCM hermaphrodites. RNAi was preformed using the empty pL4440 or pL4440 expressing *odr-10* extracted from the Ahringer library and re-transformed into RNAi competent OP50 bacteria [51]. All raw data is available in **S8 Table**.

### RNA extraction, library preparation, RNA sequencing, and analysis

For hermaphrodites, 50–100 gravid adults were allowed to lay eggs for 6 hours on a 9 cm plate seeded with *E. coli OP50*. At day 1 of adulthood, ~ 2500 worms, per sample, were picked and washed of residual bacteria with 5 M9 buffer washes. For males, ~ 2500 L4 stage animals were picked onto fresh plates and harvested the following day. After the final wash, worms were pelleted with TRIzol flash frozen in liquid nitrogen and subject to 20–25 freeze-thaw cycles to break open the cuticle. After the final thaw, supernatants were transferred and RNA was extracted using a Direct-zol RNA Microprep Kit (Zymo Research) in accordance with the manufacturer’s instructions. RNA quality and yield was measured using a Nanodrop spectrophotometer.

RNA samples were sequenced by Novogene. Messenger RNA was purified from total RNA using poly-T oligo-attached magnetic beads. After fragmentation, the first strand cDNA was synthesized using random hexamer primers followed by the second strand cDNA synthesis. The library was ready after end repair, A-tailing, adapter ligation, size selection, amplification, and purification. The library was checked with Qubit and real-time PCR for quantification and bioanalyzer for size distribution detection. Sequencing was performed using an Illumina HiSeq 2000 with paired-end reads of 150 bp, averaging over 20 million reads per sample.

All data processing procedures were conducted using Galaxy V. 24.1.0. Adapter trimming and filtering of low-quality reads were performed with Trimmomatic [52]. Trimmed reads were mapped to the *C. elegans* genome (WBCel235) using HISAT2 v. 2.2.1 [53] with default settings, and quantified using featureCounts v. 2.0.1 [54]with default settings. Differential expression analysis was performed using DESeq2 v. 1.48.1 [55] for R v. 4.5.0. Genes with log2 fold-changes <−0.6 or > 0.6 and FDR-adjusted p-values < 0.05 were considered differentially expressed.

k-means clustering was performed with the normalised and scaled read (counts per gene) matrix using the base-R function *kmeans*. The optimal value of k was determined using the Silhouette method (ref). Gene set enrichment analysis was performed using the *C. elegans*-specific web-based tool WormCat (ref) with a Bonferroni-adjusted p-value cutoff of 0.05. Tissue enrichment analysis was performed using the tissue enrichment analysis tool included in the WormBase tool suite (ref) with an FDR-adjusted p-value cutoff of 0.05.

cDNA synthesis and RT-qPCR. 600–800 day 1 adults were collected, rinsed washed twice in M9 buffer and allowed to settle by gravity. 200 µl TRIzol was added to the pellet before flash freezing in liquid nitrogen and storage at -80°C. Worms were dissociated by ~6 freeze–thaw cycles, followed by a further 4 cycles incorporating a grinding step (10 cycles total). Total RNA was extracted using the Direct-zol RNA Miniprep Kit (Zymo Research) including on-column DNase I treatment, and quantified by Nanodrop. First-strand cDNA was synthesised from 400 ng total RNA per sample using the UltraScript cDNA Synthesis Kit (PCRBIO) in 20 µl reactions. qPCR was performed on a QuantStudio 3 (Thermo Fisher) using qPCRBIO SyGreen Mix Lo-ROX (PCRBIO). Each 10 µl reaction contained 2 × SyGreen mix, forward and reverse primers, and cDNA (diluted 1:10). Cycling conditions were 95°C for 2 min, 40 cycles of 95°C for 15 s and 65°C for 1 min, with a final melt-curve analysis (95°C for 15 s, 60°C for 1 min, and a continuous ramp to 95°C for 1 sec); all ramp rates were 1.6°C/s except the dissociation ramp (0.15°C/s). Reactions were run in technical triplicate across three independent biological replicates. Relative expression was normalised to the housekeeping gene *cyc-1* and calculated using the ΔΔCt method.

Primer sequences: *ins-8* (F: GCATGCGATTCAAATACAAAGTCGA, R: CGACATCAATGTTGTCCTTAA), *arrd-8* (F: CGCGAACAATAAAATCCCTGGC, R: GGGACGCATGGAAATGTTCG), *pqn-15* (F: CAGCAAATGCAGATGCAACAGC, R: CTGATGAGGTTGAGACGGCGG), *asp-14* (F: CCAAAGGAGGACAAACTCTGG, R: CGACCGCTGACATTGTTATTCC), *scl-14* (F: CAGCCACAAGGAAACTACTTG, R: CGTGCGAGGCCGCTACCGGAC) and *cyc-1* F:CTA AAC GTG GAT TGG CGG CC R: GCC CAT GGT AAA GCG TAC GG.

### OxoPlate assay

The oxygen consumption assays were adapted from [19,22] and conducted using a PreSens 96-well OxoPlate (O_2_ monitoring 96-well microtiter plate with O_2_ sensors at the bottom of the wells). O_2_ consumption was measured under normal conditions and after the sequential addition of electron transport chain inhibitors and a mitochondrial uncoupler. Overall mitochondrial respiration and bioenergetic health was assessed by calculating basal O_2_ consumption, ATP synthase-linked O_2_ consumption, proton leak O_2_ consumption, maximal O_2_ consumption, non-mitochondrial O_2_ consumption and spare capacity.

Basal O_2_ consumption was first measured in the absence of any inhibitors. The next step involved the addition of N, N-Dicyclohexylcarbodiimide (DCC), a complex V inhibitor, which blocks ATP Synthase and decreases O_2_ consumption. Carbonyl cyanide p-trifluoromethoxyphenylhydrazone (FCCP) was then added to disrupt the inner mitochondrial membrane gradient resulting in maximal O_2_ consumption. Finally, sodium azide, a cytochrome oxidase inhibitor, was added to completely inhibit the electron transport chain and allow assessment of non-mitochondrial (NMT) linked O_2_ consumption. ATP synthase-linked O_2_ consumption was calculated by subtracting DCC-linked O_2_ consumption from the baseline O_2_ consumption. Proton leak O_2_ consumption was calculated by subtracting NMT from DCC-linked O_2_ consumption. Maximal O_2_ consumption was calculated by subtracting NMT from FCCP-linked O_2_ consumption. Finally, spare capacity was calculated by subtracting basal O_2_ consumption from maximal O_2_ consumption.

### ATP Measurements

ATP content was measured using the CellTiter-Glo Luminescent Cell Viability Assay Kit (Promega). Flash-frozen age-matched worms were lysed in 50µL of M9 and added to an opaque 96-well plate with equal amounts of CellTiter-Glo 3D Reagent. Samples were agitated for 5 mins and incubated at room temperature for 25 mins to stabilise the luminescent signal before luminescence was recorded using the OMEGA plate reader (BMG FLUOstar).

ATP levels in the body wall muscle were measured using the Queen-2m ratio metric ATP sensory transgenic strain as described in [56].Briefly, age-matched worms were imaged using the LSM880 point scanning confocal microscope at 20X magnification with a 5x5 tile scan. Images were maximally projected using Zen Black (Zeiss) and uploaded to FIJI (Image J) for analysis. After background subtraction, the mean whole-body fluorescence for 405 nm λ_ex_ (blue channel; high ATP) and 488 nm λ_ex_ (green channel; low ATP) was measured. To calculate the 405/488 excitation ratio, the mean whole-body fluorescence at 405 nm λ_ex_ was divided by 488 nm λ_ex_ which gives a value to indicate relative ATP levels. A higher 405/488 excitation ratio indicates high ATP levels.

### Microscopy

Fluorescence/brightfield microscopy: age-matched adults were placed on a 2.5% agarose pad for imaging and immobilized with 50mM tetramisole hydrochloride. SKN-1B::GFP expression pattern was determined using NeuroPAL, a multicolour transgenic reporter which allows for nervous-system-wide neuronal identification in *C. elegans* using a combination of coloured, fluorescent reporters [25]. Male tail images were acquired on Leica DMR microscope at 63X.Confocal microscopy for mitochondrial network quantification: age-matched adults were placed on a 2.5% agarose pad for imaging and immobilized with a 50mM concentration of tetramisole hydrochloride solution. A Zeiss LSM880/Elyra/Axio Observer.Z1 confocal microscope was used to acquire the images at 40x to 63x magnification. Muscle fibres and mitochondria networks in the intestine and neurons were analysed using Fiji Image J with SQUASSH and Mitochondrial Network Analysis (MiNA) plugins [27,28].

### Behavioural assays

Exploration: were performed as in [4,15]. Briefly, 3.5 cm or 6 cm plates of 5 or 10 ml NGM agar respectively were dried for three days before seeding with 200µL or 350µL freshly prepared *E. coli* OP50 culture. Plates were tilted to ensure coverage of the entire surface and dried overnight. Single L4 animals were placed on plates and allowed to roam for 16hrs (hermaphrodites) or 4hrs (males) at 25°C. As males explore more in general compared to WTs, the exploration assay duration for males was shortened to either 1 or 4 hrs to avoid the plates becoming ‘too busy’ with tracks and difficult to score and determine differences between genotypes**.** For comparison, we show the assay at a 16 hr time points (S1 Fig). Plates were imaged using WormLab (MBF Biosciences) or an iphone attached to a dissecting microscope and superimposed with a grid and analysed using ImageJ (FIJI). Track(s) within a square was classified as one filled square, with a maximum of 109 filled squares per plate. Male leaving and staying assays: To assess a male’s tendency to leave a food source in the absence of a hermaphrodite, 9 cm plates were poured with a thin coating of NGM agar to allow visualisation of tracks. Once dried, a lawn of 100µl *E. coli* OP50 was seeded in the centre of each plate and dried overnight. Single males were placed in the middle of this food spot and examined every hr. A male was classed as ‘leaving’ if it travelled to within 1 cm of the plate boundary. The same assay was performed to assess the tendency of a male to stay on food in the presence of a hermaphrodite, except a single *unc-31* hermaphrodite was placed on the same food spot as the male. To assess male leaving behaviour in response to pheromones, *unc-31* hermaphrodites were placed on the food spot for three hours, and then removed before the male was added. Exploration in the absence of food was measured on the same plates without food spots. For RNAi experiments, animals were from from eggs on RNAi plates (NGM supplemented with 1 mM IPTG and 50 µg/mL Ampicillin) seeded with *E. coli* OP50*(xu363)* bacteria expressing double-stranded RNA targeting *odr-10* or empty vector control (L4440). Males were transferred to standard NGM spot plates as above. all these assays, plates were scored over a 12hr period at 20°C and care was taken to minimize disturbance to the plates. Animals that burrowed or did not leave the assigned boundary by the end of the assay were excluded from the final analysis. 15–20 animals were analysed per assay.

To quantify thrashing, individual males were transferred into a 50µl drop of M9 buffer on a unseeded NGM plate and allowed to acclimatize briefly. Thrashing behaviour was recorded using the WormLab imaging system (MBF Bioscience) and thrashes in 60 second counted manually. A bending of more than 50% of the animal’s body in the opposite direction was deemed a single thrash.

### Male fertility assay and spermatocyte measurements

WT and *skn-1b* males were aged up to day 5 of adulthood. These aged males were kept on individual mating plates containing 3 virgin late-L4 hermaphrodites. After 24 hours, the male was removed and hermaphrodites isolated onto individual plates. A male was scored fertile if male progeny were identified in the F1 population. N = 30. Spermaotcytes measurements were performed as previously described with minor modifications [57]. Male *C. elegans* were raised up to day 5^th^ of adulthood. On day 5^th^, 20–30 male worms were washed with 0.01% Tween PBS to remove excess bacteria, fixed in methanol for 5 min. Worms were washed once again with 0.1% Tween PBS. DAPI dye was added immediately and kept for 15 min. Worms were washed once again with 0.1% Tween PBS and mounted on 2.5% agarose pads for visualization. DIC and DAPI images were acquired using an Olympus IX 81 microscope. DAPI images were analysed in FIJI. Similar region from upper spermatheca was selected for WT and *skn-1b* animals and individual, distinct spermatocytes for each worm were counted.

## Supporting information

S1 Table Differentially expressed genes in WT *vs skn-1b* mutant hermaphrodites and males. (XLSX)

S2 Table k-means clustering using the most statistically significant DEGs derived from all possible between-group comparisons in our dataset. (XLSX)

S3 Table Gene Set Enrichment Analysis and Tissue Enrichment Analysis of DEGs from WT *vs skn-1b* mutant comparisons, hermaphrodites and males. (XLSX)

S4 Table DEGs from WT *vs skn-1b* mutant comparisons, hermaphrodites and males corresponding to known SKN-1 targets. (XLSX)

S5 Table skn-1b-dependent genes from hermaphrodites and males specifically implicated in mitochondrial function and morphology. (XLSX)

S6 Table *skn-1b*-dependent changes in mRNAs across neuron types. (XLSX)

S7 Table Genes identified as movement related are differentially expressed in *skn-1b* mutant hermaphrodites and males. (XLSX)

S8 Table All supporting raw data. (XLSX)

S1 Fig Exploration of day 1 adult males at 16 h. Representative images of plates shown.
(TIF)

S2 Fig Male tail and spermatheca in WT and *skn-1b* mutant *C. elegans.* **A)** Tail of day 5 adult male does not show significant morphological changes in WT and skn-1b mutants. Images shown were acquired at 63X. **B)** Spermatid count between day 5 adult male and *skn-1b* mutant male does not show any significant-difference. n=>30 animals per group. Statistical comparisons were made using two-tailed t-test,ns = non-significant. Individual spermatids were counted in similar per unit area (1 cm x 1 cm) of a section of spermatheca in WT and *skn-1b* animals. Images generated in Biorender agreement number UN29WARSYX http://BioRender.com/o0ejms4.
(TIF)

S3 Fig Impact of SKN-1B on mitochondria and actin in muscles. **A)** Scoring system of the expression of myo-3::mitoGFP in *C*. *elegans* body wall muscle. Mitochondria are labelled using the myo-3::GFP(mit) reporter which marks the outer mitochondrial membrane, delineating their shape and allowing their morphology to be scored as very organised (lined up) to very disorganised (fragmented pattern) as used in [4]. This is similar to a non-fragmented to fragmented score, but does not make any assumptions about the fusion or fission of the organelles. **B-C)** Images and qualitative scoring system of body wall muscle organisation in WT and *skn-1b* mutant hermaphrodites (Herndon et al., 2002).
(TIF)

S4 Fig SKN-1B does not alter whole body ATP concentration in hermaphrodites or males. **A&B)** Quantification of ATP levels in hermaphrodites and males. n = 75 animals per group. **C)** Quantification of 405/488 excitation ratio (ATP level) in hermaphrodites. n=>40 animals per group **D)** Representative images showing the expression pattern of the Queen-2m ratiometric ATP sensor in the body wall muscles of WT and *skn-1b* mutant hermaphrodites. Statistical comparisons were made using two-tailed t-test, n = non-significant.
(TIF)

S5 Fig SKN-1B is differentially expressed in hermaphrodites and males. SKN-1B::GFP expression in hermaphrodites **(A)** and male **(B)** ASI neurons. DiI staining shows structures of the chemosensory neurons. **C)** SKN-1B::GFP and NeuroPAL co-expression identify SKN-1B::GFP expression in the VA11 neuron in the male tail region. Representative images shown from analysis of >20 animals per group.
(TIF)

S6 Fig Differentially expressed genes in WT vs *skn-1b* mutants. **A)** data from RNA Sequencing of day 1 hermaphrodites. **B)** Validation of these genes with qPCR: Transcript levels were normalised to the housekeeping gene *cyc-1;* mean of three independent biological replicates. For all five genes tested, the direction of change detected by RT-qPCR is consistent with the RNA-seq data. Error bars indicate SEM.
(TIF)

S7 Fig Sex-specific transcriptional activity in WT and *skn-1b* animals. **A-B)** Volcano plots showing sex-specific gene expression patterns in WT (N2; A) and *skn-1b* (B) animals. The vertical and horizontal lines represent the LFC (<-0.6 or >0.6) and FDR-adjusted p-value (<0.05) cutoffs, respectively. Counts of up- and down-regulated genes are provided in the annotations. **C)** Comparison of male-enriched genes in WT animals derived from this study with those published in Kim et al. 2016. A more stringent Log_2_-fold-change (LFC) and p-value cutoff was applied to provide a like-for-like comparison with the published dataset (LFC > 2, FDR-adjusted p-value < 0.01). P-value annotation, hypergeometric test.
(TIF)

S8 Fig Optimal cluster determination. **A)** Average silhouette width for clusters (k) 1–10. The value of k with the highest average silhouette width is denoted with the dashed line and was used for subsequent k-means clustering. **B)** Principal components analysis of differentially expressed genes labelled by cluster membership.
(TIF)

S9 Fig Functional categorisation of movement-associated genes. **A)** Genes identified as movement related in STRING that were differentially expressed in *skn-1b* mutant males were classified into categories muscle (49), neuron (117), ECM/adhesion (60), signalling/transcription factor (197) and other (417) based on GO terms and descriptions from WormBase Simple Mine. Full list and hermaphrodite movement associated genes are in S7 Table**. B)** Overlap of genes in each category. For clarity only genes log2 fold-change >2 or <−2 are listed.
(TIF)
