# Supplementary material for: Neuronal Nrf/SKN-1B controls a sexually dimorphic neuroendocrine pathway for C. elegans exploratory behaviour
Source: PLoS Genet. 2026 Aug 13;22(8):e1012239. doi: 10.1371/journal.pgen.1012239 (PMC13492746; doi:10.1371/journal.pgen.1012239)
Supplement: Supplementary file 1 [file pgen.1012239.s018.docx]

**Additional Editor Comments:**
The following three major concerns highlighted by the reviewers need to be addressed:

**1. Behavioral assays.**
Better consistency and standardization of the assays used to measure exploration regulated by food and male searching, including stronger behavioral controls measuring basal locomotion.

*We have addressed this point in a number of ways including:* ***1)*** *standardising the exploration assays to the same time frame (within a sex),* ***2)*** *including additional food-leaving assays for older animals,* ***3)*** *including food leaving assays to further examine the interaction between skn-1b and ord-10, and* ***4)*** *by measuring basal locomotion using a thrashing assay. The details of these new data can be found in response to the individual reviewers comments below.*

**2. Mitochondrial biology**
Transform the mitochondria part into a more mechanistic section and explain how a neuronal isoform (skn-1b) controls muscle mitochondria. The part on mitochondria functions and respiration needs additional explanations and better interpretations. In Fig S3 the ATP concentrations shown are between 100-800 mM, however, many other sources have measured intracellular concentrations of ATP between 1-10 mM in eukaryotic cells. The authors should check their estimates for ATP levels in C. elegans.

*The mitochondrial function section of the manuscript has now been re-worked, taking a steer from the recommendations of reviewer 2 (which we found very helpful). In addition, we have added detail on the mitochondrial genes differentially expressed in skn-1b mutant males and provided more discussion on how a neuronally expressed transcription factor could mediate these effects.*

*We believe that the ATP measurements are correct. The assay that we used examines ATP production from 50 animals per group and is not normalised to protein concentration. Therefore the numbers are larger and we are comparing between genotypes rather than discussing specific intracellular levels.*

**3. Transcriptomic analyses**
Explain how the expression of skn-1 in ASI results in impressive sexual dimorphism in transcriptomics driving ~3k DEGs in males and ~40 in hermaphrodites. Validation of a subset of DEGs is required. The authors should also explain how the mating depends on skn-1b activities in VA11 providing rescue experiments.

*This has been addressed using a combination of additional bioinformatic analysis, data and text edits to the manuscript. As outlined in our cover letter and email correspondence, we have undertaken a significant re-analysis of the RNA Seq data. This has altered the gene numbers but has allowed for a much more comprehensive analysis – improving the content of the manuscript whilst not changing the overall findings. The specifics are below in relation to each of the reviewers points.*

*The question of whether the VA11 neuron is required for the mating phenotype was also raised by reviewer 2, although they frame it as unlikely, [“it seems unlikely that the mating phenotype depends on skn-1b function in VA11”] as there is [“no precedent for the function of that neuron in male mating”]. And suggest an alternative hypothesis [“that skn-1b mutants preserve their muscle excitability better than wildtype males”]. Therefore rather than carry out rescue experiments at this time, we have softened the link between this neuron and the behaviour phenotype and cited the additional articles suggested by reviewer 2.*

*A full summary of these changes can be found below.*

**Reviewers' comments:**

**Comments to the Authors:**

**Reviewer #1:** This is a nice piece of work reporting the different impact that loss of skn-1b has on either sex of C. elegans. Specifically, the authors show that skn-1b regulates feeding behaviour in hermaphrodites and reproductive behaviour in males. This is accompanied by altered mitochondrial function in hermaphrodites but not in males and by a drastically sexually dimorphic transcriptional change in mutants versus wildtype worms. Intriguingly, all these changes are suggested to arise from the expression of skn-1b in only one neuronal class.

Given the conservation of the gene skn-1/nrf and what little is known on the neuronal role of this pathway in mammals, this work provides new insights and data sets that will be of interest to other researchers working on aging, metabolism and sexual dimorphism. Below I outline several concerns I have which should be addressed before publication.

**Major:**

**1-** One of the main conclusions of the paper is that skn-1b acts as a switch regulating foraging in hermaphrodites and mate-searching in males. The focus is on measuring these two behaviours in terms of exploration. Although it is a little difficult to unambiguously pinpoint what a worm is exploring in search of, the community (and indeed the authors here) have come up with different assays to measure food exploration versus mate searching. While exploration regulated by food is measured as % of area travelled through on a plate either covered in food or completely empty of food, mate searching is measured as the probability of leaving a small patch of food. The authors are not consistent in the assays they use to measure the different types of exploration. They conclude that skn-1b does not regulate food exploration in males because they do not observe differences in % area explored on a lawn or a plate empty of food (Fig 1, D, E and F). Yet, they use this same assay in 1K to make a statement about the ability of ageing males to go mate searching. The authors should measure mate-searching in 1K, 5 G and H with the food leaving assay. Otherwise it is difficult to disentangle the effects of skn-1b loss in either type of exploration. In fact, an alternative interpretation is not that aging skn-1b males have lost their drive to explore in search of mates (or even of food) but simply that their locomotion is impaired in any context. Measurements of basal locomotion should be provided if the authors want to make the claim that loss of skn-1b affects mate searching specifically.

*Thank you for this feedback, we found it very helpful and in general agree with you. To add clarity to our argument we have done some extra experiments.*

***1)*** *In* ***Figure 1K****, we showed that aged skn-1b mutant males have increased exploratory behaviour compared to WT age-matched controls. We can now also show that aged skn-1b males leave food faster than WTs in search of mates. These new data are shown in* ***Figure 1N*** and commented on in the text. *Thus both the exploratory and food leaving behaviour of older skn-1b mutants is enhanced.*

*“Furthermore day-5 skn-1b mutants also retained their enhanced food-leaving behaviour (****Fig 1N****).”*

***2)*** *In Figure 5G-H, we had shown that skn-1b interacted with odr-10 to control exploratory behaviour. We have now done further experiments to test whether odr-10 interacts with skn-1 for mate searching. We find that similarly to skn-1b mutants, odr-10 RNAi increases food leaving behaviour, but that the effects of the two are not additive. This has been added as a new panel* ***(Figure 5H)*** *and addressed in the text. That section now reads as follows.*

*“Finally, we tested whether this decrease in odr-10 expression can explain the altered behavior observed in skn-1b males (****Fig 1E****). We tested the impact of odr-10 RNAi knockdown on WT and skn-1b male exploratory behavior under fed conditions. WT males fed odr-10 RNAi showed a modest but significant increase in their exploratory behaviour supporting the idea that decreasing odr-10 levels promotes exploration (****Fig 5G****). Interestingly however, although both WT and skn-1b mutant males fed on control RNAi plates had the same level of exploratory behaviour, skn-1b mutation and odr-10 RNAi were non-additive with respect to exploratory behaviour (****Fig 5G****). Moreover, similarly to skn-1b mutation ord-10 RNAi caused increased food-leaving behaviour in males, but these effects were also non-additive* ***(Fig 5H)****. Together with the expression data, these data imply that skn-1b and odr-10 interact to control exploratory behaviour in males, and drive mate searching, and that this could account for the SKN-1B mediated sexually dimorphic effects on exploratory behaviour and possibly contribute to the improved late-life mating phenotype associated with skn-1b mutation.”*

***3)*** *In addition, there is the possibility that skn-1b mutants are somehow altered in their ability to move. In Tataridas-Pallas et al., 2021 we showed that WT and skn-1b hermaphrodites had very similar movement/thrashing profiles. We have now extended this and compared thrashing activity in both WT and skn-1b males (young and aged). However we find no significant differences suggesting that the other movement phenotypes we observe are linked to behaviour rather than neuro-muscular function. These new data are shown in* ***Figure 1O*** *and addressed in the text as follows.*

*“Finally we explored the possibility is that skn-1b mutants are more active than WT animals, or display increased muscle excitability [17]. However we tested the thrashing activity of both day 1 and day 5 skn-1b mutants in liquid, and found no differences compared to WT (****Fig 1O****). This is consistent with our previous finding in skn-1b mutant hermaphrodites and suggests that the foraging and mating behaviours observed in male skn-1b mutants derive from neuronal signalling rather than a muscle phenotype [4].”*

2- **The discussion and main conclusion of what the normal function of SKN-1b is in either sex is a bit muddled up.** Figure 5 states ‘skn-1b phenotypes’ but then it depicts SKN-1b normal function, not skn-1b mutant phenotype. SKN-1b interferes with reproductive behaviour during aging since it is the loss of function mutant that is better at mate searching and mating (fig 1J and K).

*Thank you for pointing this out. The heading in Figure 5 (panel 1) has been altered to: ‘SKN-1B drives sexually dimorphic phenotypes’.*

3- The section assessing mitochondria function and respiration is quite unclear for a non-expert. Please rewrite explaining how each conclusion is drawn from the experimental manipulation. Also, unclear what is dysfunctional and what isn’t and the discussion should address differences between the sexes in this regard.

*Thank you for this feedback. We have taken the opportunity to re-work this section of the results and believe that it is now clearer and explain the sex-specific differences. For ease of reviewing we have pasted this below.*

*“To determine whether these morphological changes impact mitochondrial function in either sex, we measured respiratory activity using PreSens OxoPlates® as described in [19] under normal condition and in the presence of electron transport chain inhibitors (****Fig 2E****) [19]. Under normal conditions, hermaphrodite skn-1b mutants exhibited significantly elevated OCR (36% increase compared to WT, p=<0.001), indicating increased respiratory activity (****Fig 2F).*** *To assess ATP-linked respiration, we inhibited ATP synthase using N,N-Dicyclohexylcarbodiimide (DCC). Following DCC treatment, skn-1b mutant hermaphrodites also showed a 33% increase in OCR relative to WT (****Fig 2F****).*

*Sequential treatment with carbonyl cyanide-p-trifluoromethoxyphenylhydrazone (FCCP) and sodium azide was used to assess maximal and non-mitochondrial respiration, respectively. The non-mitochondrial OCR was significantly increased in skn-1b mutant hermaphrodites (52% increase vs WT;* ***Fig. 2F****), indicating elevated background oxygen consumption. Despite these increases, skn-1b mutant hermaphrodites displayed no significant differences in proton leak, ATP-linked respiration, maximal respiration, or spare respiratory capacity compared to WT (****Fig. 2G–I****). Together, these findings indicate that although basal respiration is elevated, the overall capacity of mitochondria to respond to energetic stress is preserved [20].*

*Consistent with this, whole-body and muscle-specific ATP levels were unchanged in skn-1b mutants relative to WT (****Fig. S4A-C****). The combination of increased oxygen consumption with unchanged ATP levels suggests reduced coupling efficiency, such that mitochondria require increased respiration to maintain normal ATP production. This is consistent with the observed mitochondrial morphological defects and indicates functional inefficiency rather than a loss of respiratory capacity.*

*In contrast, skn-1b mutant males showed no significant differences in basal OCR or in response to ATP synthase inhibition compared to wild type (****Fig. 2J****), indicating that respiratory activity is largely unaltered under these conditions. However, the non-mitochondrial OCR was significantly reduced in mutant males (25% decrease vs WT; p < 0.05;* ***Fig. 2J****), suggesting a lower level of background oxygen consumption and a potentially improved bioenergetic profile [20]. Further analysis revealed that skn-1b mutant males exhibited a marked increase in proton leak (~120% increase vs WT), while maximal respiration, spare respiratory capacity, and ATP-linked respiration remained unchanged (****Fig. 2G–I****). Although increased proton leak typically reflects reduced coupling efficiency, the absence of changes in ATP levels (****Fig. S4C-D****) and other respiratory parameters suggests that overall mitochondrial function is maintained [20–22].*

*Taken together, these data reveal a clear sex-specific role for SKN-1B in mitochondrial regulation. In hermaphrodites, loss of SKN-1B leads to increased basal respiration and reduced coupling efficiency, consistent with functionally compromised mitochondria despite preserved respiratory capacity. In contrast, males exhibit largely intact mitochondrial function, with only selective changes in proton leak and non-mitochondrial respiration. These findings suggest that SKN-1B is required to maintain efficient mitochondrial function specifically in hermaphrodite muscle, but is largely dispensable in males.”*

*In addition, we added the following to the legend of Figure 2 to explain how different measurements were calculated.*

*“****E)*** *A representative O_2_ consumption curve generated from a respirometry experiment using PreSens OxoPlates® (ref). Basal O_2_ consumption (OCR) is measured in normal conditions and in response to mitochondrial inhibitors. These values are used to calculate parameters of respiratory function: ATP synthase-linked respiration, proton leak, maximal (i.e. uncoupled) respiration and the mitochondrial spare capacity, and non-mitochondrial respiration. ATP-linked respiration = basal OCR – OCR after ATP synthase inhibition (DCC); proton leak = OCR after ATP synthase inhibition - non-mitochondrial OCR; maximal respiration = OCR after FCCP uncoupling; spare respiratory capacity = maximal – basal OCR; non-mitochondrial OCR = OCR after complete ETC inhibition (sodium azide).* *”*

*And the following was re-worked in the discussion section to clarify the impact of the sex-specific phenotypes we observe.*

*“We also show that the altered mitochondrial networks in skn-1b mutant hermaphrodite muscles correlates with changes in mitochondrial function consistent with inefficient respiratory activity and hallmarks of a low bioenergetic health index (****Fig 3E-I****) [20]. However, the essentially ‘normal’ state of skn-1b mutant male mitochondria suggests that sexual dimorphism is an important consideration when studying mitochondrial dynamics. Dysregulated mitochondrial structures or function is complex, and a naive interpretation would be that dysregulation is metabolically bad for an organism and in this scenario that skn-1b mutant hermaphrodites are at a disadvantage compare to males. However, modulating mitochondrial proton leak is a therapeutic target for complex metabolic diseases including obesity and diabetes in humans, with a higher proton leak correlating to a higher basal resting metabolic rate, energy expenditure, and weight loss [46]. Further studies are required to understand the physiological impact of these sex-specific effects.*

*It is counterintuitive that the changes in mitochondrial morphology and function are not mirrored by changes in mitochondrial gene expression in hermaphrodite animals. Instead, skn-1b mutant males, where mitochondrial phenotypes are essentially normal exhibit changes in mitochondrial-associated gene expression (****Table S5****). The involvement of some of these in mitochondrial morphology or metabolism could suggest a neuronally driven programme of gene expression that compensates for SKN-1B driven effects in a mutant background.”*

4- While possible, it is a little surprising that expression of skn-1 in just ASI (in both sexes) leads to the massive sexual dimorphism in transcriptional profile. Given that the authors do not provide any rescue experiments or CRISPR-based reporter expression, they should address such limitations of their study in the discussion. Equally, it seems unlikely that the mating phenotype depends on skn-1b function in VA11 in the absence of rescue experiment and no precedent for the function of that neuron in male mating. An alternative interpretation is that skn-1b mutants preserve their muscle excitability (which is known to affect mating success in ageing males -work from Rene Garcia’s lab) better than wildtype males.

*We agree that it would be helpful to make the limitations clearer. The following has been added/altered in the discussion section.*

*“Our data shows a striking, and surprising, difference in skn-1b-dependent transcriptional change between males and hermaphrodites…..*

*……VA11 forms neuro-muscular junctions with the body wall muscles and is implicated in locomotion, specifically backwards movements [39]. Although not directly linked to male mating, it is connected to the post cloacal sensilla neurons (PDB, PDC or PVV) which are responsible for controlling movement related to copulation, and the hook sensillum neurons (HOA and HOB) which are important for vulva sensing – although ablation of this neuron did not cause any mating-related phenotypes [26,27]”.*

*To acknowledge the Garcia’a lab contribution to this field, we have now cited their work in relation to muscle excitability and mating success. However, we do not think that the late-life mating success of skn-1b mutants is due to increased muscle excitability as their general locomotion is unchanged compared to WT in a thrashing assay. These experiments were carried out thanks to comments by both reviewers and have now been included in* ***Figure 1O****. The following has been added to the results section.*

*“Finally we explored the possibility is that skn-1b mutants are more active than wild-type (WT) animals, or display increased muscle excitability [17]. However, we tested the thrashing activity of both day 1 and day 5 skn-1b mutants in liquid, and found no differences compared to WT (****Fig. 1O****). This is consistent with our previous finding in skn-1b mutant hermaphrodites [4].”*

Minor points:

1- In sentence in first paragraph of results “Hermaphrodite C. elegans alternate between three behavioural states: food searching, food consumption, and satiety-induced quiescence “ please add that this is in the context of foraging specifically, since worms display many other states that those 3

*This now reads:*

*“In relation to foraging, hermaphrodite C. elegans alternate between three behavioural states: food searching, food consumption, and satiety-induced quiescence [13].”*

2- Regarding mitochondria organised or disorganised - should elaborate a little more on how they quantify this. What does organisation entail and how does it differ from fragmentation

The following has been added to the legend of **Figure S2A**.

*“Scoring system of the expression of myo-3::mitoGFP in C. elegans body wall muscle. Mitochondria are labelled using the myo-3::GFP(mit) reporter which marks the outer mitochondrial membrane, delineating their shape and allowing their morphology to be scored as very organised (lined up) to very disorganised (fragmented pattern) as used in [4]. This is similar to a non-fragmented to fragmented score, but does not make any assumptions about the fusion or fission of the organelles.”*

3- Indicate in the figure of graphs 2C, D, F, J whether data is for males or hermaphrodites

*Symbols for hermaphrodites and males have now been added to these figure panels.*

4- The references implicating Daf-7 and insulin in male mate-searching behaviour are Hilbert et al and Lipton et al, respectively. Please add

Hilbert, Z. A. & Kim, D. H. Sexually dimorphic control of gene expression in sensory neurons regulates decision-making behavior in C. elegans. Elife 6, 386.
Lipton, J., Kleemann, G., Ghosh, R., Lints, R. & Emmons, S. W. Mate Searching in Caenorhabditis elegans: A Genetic Model for Sex Drive in a Simple Invertebrate. J Neurosci 24, 7427–7434 (2004).

*Thank you, this was an oversight on our part. These references have been added in at the end of this sentence.*

*“Additionally, the expression of odr-10 a seven transmembrane domain food chemosensory receptor, that controls male feeding and mate searching behaviours, downstream of the TGF-β and insulin signalling was also down-regulated Log_2_ (fold Change) -3.09 p=<0.05) in skn-1b mutant males (****Fig. 4A****)[14,39–41].”*

5- When bringing up odr-10, Ryan et al reference should be included

Ryan, D. A. et al. Sex, age, and hunger regulate behavioral prioritization through dynamic modulation of chemoreceptor expression. Curr. Biol. 24, 2509–2517.

*Thank you, again this was an oversight on our part. This reference have been added in at the end of this sentence.*

*“This decreased expression of odr-10 promotes mate-searching behavior [14,42].”*

--------------------------------------------------------------------------------------------------

**Reviewer #2:** Summary
This work investigates the role of neuronal skn-1b in regulating sexually dimorphic behavior in C. elegans. The authors report that skn-1b mutants display enhanced mate-searching and improved late-life mating efficiency in males, as well as hermaphrodite-specific changes in mitochondrial morphology. Bulk RNA-seq reveals substantial male-biased transcriptomic changes, with ~3,000 DEGs in males vs only 44 in hermaphrodites. The authors propose a model in which neuronal SKN-1B modulates daf-7/TGF-β, insulin signaling, and odr-10 expression to influence male exploratory/mate-searching behavior, and potentially mitochondrial dynamics through an adjacent pathway.

Overall Impression
The study addresses an interesting question regarding sex-specific neuronal and behavioral regulation, which is an emerging topic in nematode biology and relevant across species. The manuscript integrates transcriptomics, behavioral assays, and imaging, and the male skn-1b RNA-seq dataset will likely be a useful community resource. With additional functional data, deeper transcriptomic analysis, and improved figure clarity, the work could make a meaningful contribution. At this stage, I would consider it a major revision since there weren’t fatal flaws, but more so missing data to back the interpretation.

**Main Concerns**
A. **Behavioral Interpretation & Assays**
• Assay timescales are quite different (e.g., male exploration on food for 4 hrs vs ~12 hrs off food). Given that skn-1b males begin leaving food at ~4 hrs based on assay fig 1, **standardizing assay windows would strengthen interpretation**.

*We agree that standardising assays would be helpful – but there are some challenges with this. The reason that the male exploration assay is done for 1 or 4hrs, is that if you leave males on plates for longer, there are so many tracks that there is no room for interpretation. This was reported in Tataridas-Pallas et al., 2021 but to make this point clearer, and to help others wanting to do similar experiments, we have now provided supplementary data showing WT vs skn-1b mutant male exploration at 16hrs. These data have been included in* ***Figure S1****.* *Moreover, we have now included additional data for male exploration, so that both 1hr and 4hr timepoints are included, throughout the paper. These new data are in* ***Figures 1E-F*** *and* ***Figures 1L-M****.* *These new data are referred to in the legend to Figure 1 and in the methods as follows.*

***“C)*** *Exploration assay. Plates with single hermaphrodite animals are incubated for 16hrs; Males are incubated for 1 or 4 hrs (see methods and* ***Fig S1****).”*

*“As males explore more in general compared to WTs, the exploration assay duration for males was shortened to either 1 or 4 hrs to avoid the plates becoming ‘too busy’ with tracks and difficult to score and determine differences between genotypes****.*** *For comparison, we show the assay at a 16 hr timepoints (****Fig. S1****).”*

• The authors show late-life mating efficiency and maintained exploratory behavior in day 5 skn-1b males. It would be more informative to repeat the food-leaving assay at day 5 rather than exploration alone, as leaving more directly reports mate-searching prioritization.

*This is a very nice suggestion, and it was also mentioned by reviewer 1. We have now done this and find that even aged (day 5 adult) skn-1b mutant males leave food faster than WT. This further supports our conclusions that skn-1b acts in males to promote late-life mating behaviour. These data are shown in* ***Figure 1N*** *and are referenced in the results section as follows.*

*“Furthermore day-5 skn-1b mutants also retained their enhanced food-leaving behaviour (****Fig 1N****). This suggests that skn-1b mutation confers an advantage for male mate-searching behaviour in old age.”*

• **A locomotion control** would help confirm that skn-1b effects are specific to mating motivation rather than general hyperactivity.

*Again, a very sensible suggestion, that we have taken on board. It was also picked up by reviewer 1. We find that the thrashing activity of skn-1b mutant males is unchanged compared to WT on either day 1 or day 5 of adulthood. This suggests that skn-1b behavioural phenotypes are not due to general hyperactivity. These data are now shown in* ***Figure 1O*** *and is explained in the results section.*

*“Finally we explored the possibility is that skn-1b mutants are more active than wild-type (WT) animals, or display increased muscle excitability [17]. However, we tested the thrashing activity of both day 1 and day 5 skn-1b mutants in liquid, and found no differences compared to WT (****Fig 1O****). This is consistent with our previous finding in skn-1b mutant hermaphrodites [4].“*

• For the odr-10 RNAi experiment (Fig 4G), odr-10 is already very low in males, so additivity would be hard to detect. It’s also unclear how to interpret the negative result given that WT vs skn-1b males do not differ in exploration to begin with. Running the assay under starvation, or using a spot-leaving paradigm, may reveal a clearer epistatic relationship. Clarifying the RNAi exposure timing would also help.

*We agree that this is a challenging experiment. We attempted a spot leaving assay under these same conditions and found that similarly to skn-1b mutation, odr-10 RNAi causes faster food leaving in WT animals. However, the effects of skn-1b mutation and odr-10 RNAi are non-additive for food-leaving, supporting a model whereby skn-1b and odr-10 interact to control exploratory and mate searching behaviour in males. This has been added as a panel* ***(Figure 5H)*** *and addressed in the text. That section now reads as follows.*

*“Finally, we tested whether this decrease in odr-10 expression can explain the altered behavior observed in skn-1b males (****Fig 1E****). We tested the impact of odr-10 RNAi knockdown on WT and skn-1b male exploratory behavior under fed conditions. WT males fed odr-10 RNAi showed a modest but significant increase in their exploratory behaviour supporting the idea that decreasing odr-10 levels promotes exploration (****Fig 5G****). Interestingly however, although both WT and skn-1b mutant males fed on control RNAi plates had the same level of exploratory behaviour, skn-1b mutation and odr-10 RNAi were non-additive with respect to exploratory behaviour (****Fig 5G****). Moreover, similarly to skn-1b mutation ord-10 RNAi caused increased food-leaving behaviour in males, but these effects were also non-additive* ***(Fig 5H)****. Together with the expression data, these data imply that skn-1b and odr-10 interact to control exploratory behaviour in males, and drive mate searching, and that this could account for the SKN-1B mediated sexually dimorphic effects on exploratory behaviour and possibly contribute to the improved late-life mating phenotype associated with skn-1b mutation.”*

• **Overall, stronger behavioral controls would help support the neuroendocrine model (skn-1b → daf-7 → odr-10), or alternatively the claims linking skn-1b to odr-10 could be softened.**

*We feel that the additional locomotion and behavioural data provided in response to the points above helps with the claims that we make.*

B. **Mitochondrial Biology**
• The mitochondrial section shows interesting hermaphrodite-specific changes (structured networks, altered respiration) but is not well integrated with the neuronal skn-1b story. The authors do not discuss how a neuronal isoform controls muscle mitochondria, nor whether this impacts behavior or energetics.

*We hope that overall the mitochondrial narrative in the manuscript is now stronger. To further address this point, we have also added the following to the discussion section.*

*It is counterintuitive that the changes in mitochondrial morphology and function are not mirrored by changes in mitochondrial gene expression in hermaphrodite animals. Instead, skn-1b mutant males, where mitochondrial phenotypes are essentially normal exhibit changes in mitochondrial-associated gene expression (****Table S5****). The involvement of some of these in mitochondrial morphology or metabolism could suggest a neuronally driven programme of gene expression that compensates for SKN-1B driven effects in a mutant background.”*

• fzo-1 and drp-1 are DEGs in the male dataset, suggesting possible changes in fission/fusion, which could provide mechanistic insight if explored. Section feels descriptive rather than mechanistically linked.

*We agree that these are interesting genes, particularly as mitochondrial dynamics play a role in skn-1-mediated hermaphrodite behaviour (Tataridas-Pallas et al., PLoS Gen. 2021). These genes are involved in promoting mitochondrial fusion, therefore it is possible that the small increases in expression of these genes could be implicated in promoting the essentially ‘normal’ mitochondrial phenotype in skn-1b males. However, although eat-3 (2LFC=1.02) and drp-1 (2LFC=1.28) are differentially regulated in skn-1b mutant males, the fold changes are very small and we decided not to pursue it in the context of this work.*

*To provide more credence to the mitochondrial angle of this study, we extracted information from both GO analysis and STRING data sets to categorise the skn-1b-dependent changes in mitochondrial-related genes in more depth. Detail on the names and roles of these genes are now in* ***Figure S6****. The following text has also been added to support this.*

*“The enrichment of several mitochondrial terms, in particular mitochondrial metabolism, in cluster 1 supports our mitochondrial function and morphology data* ***(Fig 3E-F)****. To further explore the role of SKN-1B in mitochondrial function, we identified skn-1b-dependent genes specifically implicated in mitochondrial function and morphology using a combination of Simplemine and the STRING data base. In hermaphrodites we only identified 5 DEGs with mitochondrial roles, but in males there were 86 (****Table S5****). This latter group included genes implicated in the Electron Transport Chain (ETC), tricarboxylic acid (TCA) cycle, mitochondrial morphology and metabolism. This included subtle upregulation of well characterized genes implicated in mitochondrial fission (drp-1 L2FC 1.28) and fusion (eat-3 L2FC 1.02) but only 9/86 mitochondrial genes were altered >2 L2FC. Thus, we suggest that the magnitude of these changes is unlikely to be sufficient to account for the functional mitochondrial changes in skn-1b mutant males, and given that skn-1b mutant males exhibit ‘normal’ looking mitochondrial structures, that this is the result of a broader compensatory response.”*

C. **Transcriptomic Analysis**

*As part of the additional work done on the m/s, we undertook a new bioinformatic analysis of the data set. This included a re-analysis of the hermaphrodite and male WT vs skn-1b mutant samples to obtain DEGs, as well as a K-means clustering, gene set enrichment and tissue enrichment analysis. This has been supplemented with additional GO analysis of mitochodniral and movement genes as well as additional mining of neuronally expressed genes using cenGEN. None of the original conclusions are altered, but this new analysis provides a more thorough overview of our data. It is presented in Figure 3A-F, Figures S6-9 and Tables S1-S7 and we have reworked the entire transcriptomic section of the results.*

*To answer your specific questions below:*

• The dataset is rich but underutilized for mechanistic insight. The authors should discuss why a neuron-specific isoform would drive ~3,000 DEGs in males and only 44 in hermaphrodites.

*As a result of this re-analysis, we now find 699 genes to be differentially regulated in the skn-1b mutant hermaphrodites and 7916 in the males. Previously we had only focused on genes altered more than 2 L2FC, but now we include all above 0.6 that are statistically significant. However, the fact remains that there are far fewer changes in the skn-1b hermaphrodites compared to the males. It is possible however, that these transcriptional changes do not reflect what occurs at the protein level. We are keen to explore this in the future, but it is beyond the scope of this study. This point is addressed in our discussion.*

*“The limited transcriptional change in hermaphrodites suggests either that SKN-1B acts more subtly in modulating their metabolic processes, potentially prioritizing reproductive energy efficiency, or that SKN-1B controls protein level changes to mediate its phenotypes, which are not picked up using RNA Sequencing.”*

• The authors note up-regulation of pkd-2 and lov-1 in skn-1b males in the discussion; both have established roles in mating. Highlighting these and other male-behavoral genes in their analysis would strengthen the connection to phenotype.

*We have now included two new analysis that include these genes as well as others implicated in behavior. We now show:* ***1)*** *the neuronal expression pattern of all skn-1b DEGs (male and hermaphrodite), with male neurons implicated in mating behavior annotated (****Table S6****);* ***2)*** *the ASI expressed genes in our skn-1b DEGs (supporting the tissue enrichment analysis) and making the point that SKN-1B may control transcription both cell-autonomously and non-autonomously (****Table S3)****; and* ***3)*** *a list of all skn-1b DEGs genes annotated as ‘movement’ – a term likely to be linked to exploratory behavior (****Table S7*** *and* ***Figure S8A-B****). These points are covered in the text as follows:*

***1)*** *“To understand the impact of SKN-1B in male mating behaviour, we mined ceNGen for the precise neuronal expression pattern of skn-1b-dependent transcripts. This identified skn-1b-dependent changes in mRNAs across inter, motor and sensory neuron types, some of which are shared between hermaphrodites and males and some that are male specific (****Table S6****). Indeed, removal of skn-1b causes changes in genes expressed in neurons that have been implicated in ascaroside pheromone detection (e.g. ADF, ASH, AWA), mate finding and searching (e.g. CEM, PHA, PHB), vulval location (e.g. HOB, PCA, PCC), sperm ejaculation (SPD, SPV) and other aspects of precise mating behavior (e.g. PHD, PVD, PVV, PVX, PVY) [12,33] . This included changes in the well-characterized genes lov-1 and pkd-2; two polycystins associated with male chasing, vulval recognition and contact, in CEM, HOB and R8B neurons [34–36]. This suggests that SKN-1B function is imbedded in a variety of male mating behaviors across several neuronal classes and sub classes.”*

***2)*** *“This analysis did not identify any DEGs in the ASI neurons. However, comparison of our data set with genes identified as ASI enriched in CeNGEN (2055 in hermaphrodites and 1689 in males), 611 and 391 overlap with our sex-specific skn-1b DEGs respectively (****Table S3****). Taken alongside our k-means clustering analysis, this indicates that SKN-1B regulates transcriptional activity in a wide range of body systems in both hermaphrodites and males, and could act either cell autonomously or non-autonomously.”*

***3)*** *“To gain further insight as to whether any skn-1b-dependent transcriptional changes could account for the sexually dimorphic differences in exploratory behaviour that we observed, we cross referenced our gene lists with those assigned terms linked to exploratory behaviour e.g. ‘movement variants’ in the STRING database. In hermaphrodites there were 11 genes in this category but in the males, this provided a list of 656 genes, 500 which were up-regulated and 156 down-regulated in skn-1b mutants (****Table S7****). These genes could be categorized further into those implicated in muscle or neuronal function, extracellular matrix or adhesion or signaling, although some genes crossed into more than one category (****Fig S8A-B)****. Interestingly, this analysis showed skn-1b-dependent changes in the TGF-β and Insulin signaling pathways (****Fig 4A****), both of which have previously been implicated in male mate searching behaviors [4]……”*

• Additional mining of tissue/neuronal expression databases would improve insight. For example, str-148 is downregulated in both sexes in skn-1b mutants and is AWA-specific, with reports of expression in male CP9 neurons, which could plausibly contribute to sensory/mating phenotypes.

*The neuronal expression pattern of all skn-1b-dependent male transcripts including neurons implicated in male mating behaviours are presented in* ***Table S6*** *see above for text.*

Baseline WT male vs WT hermaphrodite comparisons for skn-1b-dependent genes would help contextualize the male-biased DEG profile.

*Our study now includes a comparison of WT and skn-1 sex-specific changes. We found that 8035 genes were sex- dependent in WTs and 11475 were sex-dependent in skn-1b mutants. These data have been shown using K-means clustering (****Figure 3D****) and discussed in the text as follows:*

*“To gather a comprehensive picture of the effects of skn-1b on the transcriptomes of animals of both sexes, we performed k-means clustering with a subset of the most statistically significant DEGs derived from all possible between-group comparisons in our dataset (****Fig 3D****). A total of 1137 genes were selected, and clustering performed on the normalised and scaled read matrix (****Table S2****), and the optimal number of clusters was determined using the Silhouette Method [28](****Fig S7A-B****). Three clusters were identified which best captured the transcriptional variation in the gene set (****Fig 3E****): a 339-gene cluster with higher expression in hermaphrodites of both genotypes (cluster 1), and two clusters with diverging expression patterns in WT and skn-1b males (clusters 2 and 3, with 241 and 557 genes, respectively). Cluster 1 also showed slight differences in activity between skn-1b and WT males, but no cluster clearly differentiated skn-1b and N2 samples across both sexes or in hermaphrodites alone, further indicating that the effects of skn-1b on the C. elegans transcriptome are highly sexually dimorphic and largely limited to males.”*

• **Validation of a subset of DEGs (qPCR, reporter, behavioral experiments) would substantially strengthen confidence**.

*In addition to the daf-7::venus reporter validating the upregulation of this daf-7 gene in skn-1b mutant males we have now included qPCR data validating the expression changes in 5 additional skn-1b-reguated transcripts. These data are presented in* ***Figure S6*** *and referred to in the* ***legend of Figure 3****.*

**Minor Concerns / Edits**
• Comment on other skn-1 isoforms and whether they were tested in males.

*The following has been added to the discussion to make reference to this point.*

*“The sex-specific roles of other SKN-1 isoforms has not been explored, but given their broad functionality and our findings here, this may offer opportunities for further discovery.”*

• It would be helpful to compare hermaphrodite vs male skn-1b expression levels in WT and mutants.

*While a comparison of sex-specific skn-1b::GFP in hermaphrodites and males could provide additional insight we have not performed these additional experiments and our RNA Seq data does not show isoform-level expression. However, he have examined sex-specific expression of skn-1 in our data set and address this in the text.*

*“We also checked whether SKN-1 mRNA was altered in skn-1b mutants and found it to be elevated in both males and hermaphrodites (both LFC > 0.96), though in neither case was this statistically significant (both p>0.083).”*

• **Include hermaphrodite off-food exploration data** or reference if previously published.

*We have not tested hermaphrodite exploration off-food. It was only done in the context of males to discriminate between food and mate searching. Therefore we have not expanded this analysis to hermaphrodites in this study.*

• Please correct for accuracy: hermaphrodite reproduction is limited by self-sperm depletion, not oocyte number.

*This has been corrected and now reads:*

*“While the hermaphrodite reproductive period is limited by self-sperm depletion and restricted to approximately 3 days…”*

• **Venn diagrams should be drawn correctly** (proportional circles and overlaps, not one size for all)

*The Venn diagram of DEGs in the main figure has now been replaced by the numbers of up and down-regulated genes on the volcano plots* ***Figure 3B****.*

• Fig notes:
o S2B–C: **images of male actin filaments would be informative.**

*We have not examined actin organisation in males. It was only done in the context of hermaphrodites to confirm that the disorganisation of the mitochondria was not due to disorganised actin. As the skn-1b male mitochondrial organisation is ‘normal’, we have not expanded this analysis to males in this study.*

o Fig 2E: add title/indicator that this assesses mitochondrial function.

*This has been added.*

o Clarify Fig 2F (herm) vs Fig 2J (male) in figure/legend.

*Symbols for hermaphrodite and male have been added to these figures.*

o Fig 3A–B: consider matching PCA/venn color schemes. A bar plot may be clearer than a venn diagram.

*Figure 3 has now been completely re-made I we hope that it is now much easier to understand.*

o Fig 3C: could **highlight male-behavior genes** and/or show absence in herm dataset.

*We have added new data showing the male behaviour genes that we identified. This is shown in* ***Figure S8*** *and in* ***Table S7****.*

o Fig 3D–E: not cited?

*This figure has been re-made and now cited correctly in the text.*

*.*

o Several references to Fig 5 appear to refer to Fig 4.

*This has been corrected.*

o Fig 6 (model): changes to the directional arrows for male phenotypes (mate-searching ↑, mating success ↑) and herm phenotypes (exploration ↓) would perhaps make more sense.

*We realise that this model was confusing. We have added the text ‘SKN-1B driven phenotypes’ to indicate that we are illustrating what SKN-1B is doing in a normal situation.*

• Methods need more detail on: genotypes sequenced, how males were generated, and genotype of worms used for mating assays.

*The following has been added to the methods.*

*“All WT vs skn-1b mutant experiments compared N2 CGCM and skn-1b(tm4241). Males were isolated from the CGCM strain and maintained by crossing with CGCM hermaphrodites.”*

• Colors are confusing- **use separate colors rather than slightly different shades** for more clarity.

*We agree that the shades of red and pink used were not ideal. This has been changed in the latest version.*

---------------------------------------------------------------------------------------------------------------------------

**Journal Requirements:**

1) We ask that a manuscript source file is provided at Revision. Please upload your manuscript file as a .doc, .docx, .rtf or .tex. If you are providing a .tex file, please upload it under the item type u2018LaTeX Source Fileu2019 and leave your .pdf version as the item type u2018Manuscriptu2019.

2) Please provide an Author Summary. This should appear in your manuscript between the Abstract (if applicable) and the Introduction, and should be 150-200 words long. The aim should be to make your findings accessible to a wide audience that includes both scientists and non-scientists. Sample summaries can be found on our website under Submission Guidelines:
https://journals.plos.org/plosgenetics/s/submission-guidelines#loc-parts-of-a-submission

3) Please upload all main figures as separate Figure files in .tif or .eps format. For more information about how to convert and format your figure files please see our guidelines: 
<https://journals.plos.org/plosgenetics/s/figures>

4) We notice that your supplementary Figures are included in the manuscript file. Please remove them and upload them with the file type 'Supporting Information'. Please ensure that each Supporting Information file has a legend listed in the manuscript after the references list.

5) When completing the data availability statement of the submission form, you indicated that you will make your data available on acceptance. We strongly recommend all authors decide on a data sharing plan before acceptance, as the process can be lengthy and hold up publication timelines. Please note that, though access restrictions are acceptable now, your entire data will need to be made freely accessible if your manuscript is accepted for publication. This policy applies to all data except where public deposition would breach compliance with the protocol approved by your research ethics board. If you are unable to adhere to our open data policy, please kindly revise your statement to explain your reasoning and we will seek the editor's input on an exemption. Please be assured that, once you have provided your new statement, the assessment of your exemption will not hold up the peer review process.

6) Please amend your detailed Financial Disclosure statement. This is published with the article. It must therefore be completed in full sentences and contain the exact wording you wish to be published.
- State the initials, alongside each funding source, of each author to receive each grant. For example: "This work was supported by the National Institutes of Health (####### to AM; ###### to CJ) and the National Science Foundation (###### to AM)."
- State what role the funders took in the study. If the funders had no role in your study, please state: "The funders had no role in study design, data collection and analysis, decision to publish, or preparation of the manuscript.".
If you did not receive any funding for this study, please simply state: u201cThe authors received no specific funding for this work.u201d
