# Supplementary material for: Neuronal Nrf/SKN-1B controls a sexually dimorphic neuroendocrine pathway for C. elegans exploratory behaviour
Source: PLoS Genet. 2026 Aug 13;22(8):e1012239. doi: 10.1371/journal.pgen.1012239 (PMC13492746; doi:10.1371/journal.pgen.1012239)
Supplement: Peer review history

# Peer review: Decision Letter 0

**DOI:** 10.1371/journal.pgen.1012239.r001

**Authors:** Monica P. Colaiácovo, Benjamin Podbilewicz

2 Feb 2026

PGENETICS-D-25-01362

Neuronal Nrf/SKN-1B controls a sexually dimorphic neuroendocrine pathway for C. elegans exploratory behaviour.

PLOS Genetics

Dear Dr. Tullet,

Thank you for submitting your manuscript to PLOS Genetics. After careful consideration, we feel that it has merit but does not fully meet PLOS Genetics's publication criteria as it currently stands. Therefore, we invite you to submit a revised version of the manuscript that addresses the points raised during the review process.

Please submit your revised manuscript by Apr 03 2026 11:59PM. If you will need more time than this to complete your revisions, please reply to this message or contact the journal office at plosgenetics@plos.org. When you're ready to submit your revision, log on to https://www.editorialmanager.com/pgenetics/ and select the 'Submissions Needing Revision' folder to locate your manuscript file.

Please include the following items when submitting your revised manuscript:

* A letter that responds to each point raised by the editor and reviewer(s). You should upload this letter as a separate file labeled 'Response to Reviewers'. This file does not need to include responses to any formatting updates and technical items listed in the 'Journal Requirements' section below.

* A marked-up copy of your manuscript that highlights changes made to the original version. You should upload this as a separate file labeled 'Revised Manuscript with Track Changes'.

* An unmarked version of your revised paper without tracked changes. You should upload this as a separate file labeled 'Manuscript'.

If you would like to make changes to your financial disclosure, competing interests statement, or data availability statement, please make these updates within the submission form at the time of resubmission. Guidelines for resubmitting your figure files are available below the reviewer comments at the end of this letter.

We look forward to receiving your revised manuscript.

Kind regards,

Benjamin Podbilewicz

Academic Editor

PLOS Genetics

Monica Colaiácovo

Section Editor

PLOS Genetics

Aimée Dudley

Editor-in-Chief

PLOS Genetics

Anne Goriely

Editor-in-Chief

PLOS Genetics

**Additional Editor Comments:**

The following three major concerns highlighted by the reviewers need to be addressed:

1. Behavioral assays.

Better consistency and standardization of the assays used to measure exploration regulated by food and male searching, including stronger behavioral controls measuring basal locomotion.

2. Mitochondrial biology

Transform the mitochondria part into a more mechanistic section and explain how a neuronal isoform (skn-1b) controls muscle mitochondria. The part on mitochondria functions and respiration needs additional explanations and better interpretations. In Fig S3 the ATP concentrations shown are between 100-800 mM, however, many other sources have measured intracellular concentrations of ATP between 1-10 mM in eukaryotic cells. The authors should check their estimates for ATP levels in C. elegans.

3. Transcriptomic analyses

Explain how the expression of skn-1 in ASI results in impressive sexual dimorphism in transcriptomics driving ~3k DEGs in males and ~40 in hermaphrodites. Validation of a subset of DEGs is required. The authors should also explain how the mating depends on skn-1b activities in VA11 providing rescue experiments.

**Journal Requirements:**

1) We ask that a manuscript source file is provided at Revision. Please upload your manuscript file as a .doc, .docx, .rtf or .tex. If you are providing a .tex file, please upload it under the item type u2018LaTeX Source Fileu2019 and leave your .pdf version as the item type u2018Manuscriptu2019.

2) Please provide an Author Summary. This should appear in your manuscript between the Abstract (if applicable) and the Introduction, and should be 150-200 words long. The aim should be to make your findings accessible to a wide audience that includes both scientists and non-scientists. Sample summaries can be found on our website under Submission Guidelines:

https://journals.plos.org/plosgenetics/s/submission-guidelines#loc-parts-of-a-submission

3) Please upload all main figures as separate Figure files in .tif or .eps format. For more information about how to convert and format your figure files please see our guidelines:

https://journals.plos.org/plosgenetics/s/figures

4) We notice that your supplementary Figures are included in the manuscript file. Please remove them and upload them with the file type 'Supporting Information'. Please ensure that each Supporting Information file has a legend listed in the manuscript after the references list.

5) When completing the data availability statement of the submission form, you indicated that you will make your data available on acceptance. We strongly recommend all authors decide on a data sharing plan before acceptance, as the process can be lengthy and hold up publication timelines. Please note that, though access restrictions are acceptable now, your entire data will need to be made freely accessible if your manuscript is accepted for publication. This policy applies to all data except where public deposition would breach compliance with the protocol approved by your research ethics board. If you are unable to adhere to our open data policy, please kindly revise your statement to explain your reasoning and we will seek the editor's input on an exemption. Please be assured that, once you have provided your new statement, the assessment of your exemption will not hold up the peer review process.

6) Please amend your detailed Financial Disclosure statement. This is published with the article. It must therefore be completed in full sentences and contain the exact wording you wish to be published.

- State the initials, alongside each funding source, of each author to receive each grant. For example: "This work was supported by the National Institutes of Health (####### to AM; ###### to CJ) and the National Science Foundation (###### to AM)."

- State what role the funders took in the study. If the funders had no role in your study, please state: "The funders had no role in study design, data collection and analysis, decision to publish, or preparation of the manuscript.".

If you did not receive any funding for this study, please simply state: u201cThe authors received no specific funding for this work.u201d

**Reviewers' comments:**

Reviewer's Responses to Questions

**Comments to the Authors:**

**Please note here if the review is uploaded as an attachment.**

Reviewer #1: This is a nice piece of work reporting the different impact that loss of skn-1b has on either sex of C. elegans. Specifically, the authors show that skn-1b regulates feeding behaviour in hermaphrodites and reproductive behaviour in males. This is accompanied by altered mitochondrial function in hermaphrodites but not in males and by a drastically sexually dimorphic transcriptional change in mutants versus wildtype worms. Intriguingly, all these changes are suggested to arise from the expression of skn-1b in only one neuronal class.

Given the conservation of the gene skn-1/nrf and what little is known on the neuronal role of this pathway in mammals, this work provides new insights and data sets that will be of interest to other researchers working on aging, metabolism and sexual dimorphism. Below I outline several concerns I have which should be addressed before publication.

Major:

1- One of the main conclusions of the paper is that skn-1b acts as a switch regulating foraging in hermaphrodites and mate-searching in males. The focus is on measuring these two behaviours in terms of exploration. Although it is a little difficult to unambiguously pinpoint what a worm is exploring in search of, the community (and indeed the authors here) have come up with different assays to measure food exploration versus mate searching. While exploration regulated by food is measured as % of area travelled through on a plate either covered in food or completely empty of food, mate searching is measured as the probability of leaving a small patch of food. The authors are not consistent in the assays they use to measure the different types of exploration. They conclude that skn-1b does not regulate food exploration in males because they do not observe differences in % area explored on a lawn or a plate empty of food (Fig 1, D, E and F). Yet, they use this same assay in 1K to make a statement about the ability of ageing males to go mate searching. The authors should measure mate-searching in 1K, 5 G and H with the food leaving assay. Otherwise it is difficult to disentangle the effects of skn-1b loss in either type of exploration.

In fact, an alternative interpretation is not that aging skn-1b males have lost their drive to explore in search of mates (or even of food) but simply that their locomotion is impaired in any context. Measurements of basal locomotion should be provided if the authors want to make the claim that loss of skn-1b affects mate searching specifically.

2- The discussion and main conclusion of what the normal function of SKN-1b is in either sex is a bit muddled up. Figure 5 states ‘skn-1b phenotypes’ but then it depicts SKN-1b normal function, not skn-1b mutant phenotype. SKN-1b interferes with reproductive behaviour during aging since it is the loss of function mutant that is better at mate searching and mating (fig 1J and K).

3- The section assessing mitochondria function and respiration is quite unclear for a non-expert. Please rewrite explaining how each conclusion is drawn from the experimental manipulation. Also, unclear what is dysfunctional and what isn’t and the discussion should address differences between the sexes in this regard.

4- While possible, it is a little surprising that expression of skn-1 in just ASI (in both sexes) leads to the massive sexual dimorphism in transcriptional profile. Given that the authors do not provide any rescue experiments or CRISPR-based reporter expression, they should address such limitations of their study in the discussion. Equally, it seems unlikely that the mating phenotype depends on skn-1b function in VA11 in the absence of rescue experiment and no precedent for the function of that neuron in male mating. An alternative interpretation is that skn-1b mutants preserve their muscle excitability (which is known to affect mating success in ageing males -work from Rene Garcia’s lab) better than wildtype males.

Minor points:

1- In sentence in first paragraph of results “Hermaphrodite C. elegans alternate between three behavioural states: food searching, food consumption, and satiety-induced quiescence “ please add that this is in the context of foraging specifically, since worms display many other states that those 3

2- Regarding mitochondria organised or disorganised - should elaborate a little more on how they quantify this. What does organisation entail and how does it differ from fragmentation

3- Indicate in the figure of graphs 2C, D, F, J whether data is for males or hermaphrodites

4- The references implicating Daf-7 and insulin in male mate-searching behaviour are Hilbert et al and Lipton et al, respectively. Please add

Hilbert, Z. A. & Kim, D. H. Sexually dimorphic control of gene expression in sensory neurons regulates decision-making behavior in C. elegans. Elife 6, 386.

Lipton, J., Kleemann, G., Ghosh, R., Lints, R. & Emmons, S. W. Mate Searching in Caenorhabditis elegans: A Genetic Model for Sex Drive in a Simple Invertebrate. J Neurosci 24, 7427–7434 (2004).

5- When bringing up odr-10, Ryan et al reference should be included

Ryan, D. A. et al. Sex, age, and hunger regulate behavioral prioritization through dynamic modulation of chemoreceptor expression. Curr. Biol. 24, 2509–2517.

Reviewer #2: Summary

This work investigates the role of neuronal skn-1b in regulating sexually dimorphic behavior in C. elegans. The authors report that skn-1b mutants display enhanced mate-searching and improved late-life mating efficiency in males, as well as hermaphrodite-specific changes in mitochondrial morphology. Bulk RNA-seq reveals substantial male-biased transcriptomic changes, with ~3,000 DEGs in males vs only 44 in hermaphrodites. The authors propose a model in which neuronal SKN-1B modulates daf-7/TGF-β, insulin signaling, and odr-10 expression to influence male exploratory/mate-searching behavior, and potentially mitochondrial dynamics through an adjacent pathway.

Overall Impression

The study addresses an interesting question regarding sex-specific neuronal and behavioral regulation, which is an emerging topic in nematode biology and relevant across species. The manuscript integrates transcriptomics, behavioral assays, and imaging, and the male skn-1b RNA-seq dataset will likely be a useful community resource. With additional functional data, deeper transcriptomic analysis, and improved figure clarity, the work could make a meaningful contribution. At this stage, I would consider it a major revision since there weren’t fatal flaws, but more so missing data to back the interpretation.

Main Concerns

A. Behavioral Interpretation & Assays

• Assay timescales are quite different (e.g., male exploration on food for 4 hrs vs ~12 hrs off food). Given that skn-1b males begin leaving food at ~4 hrs based on assay fig 1, standardizing assay windows would strengthen interpretation.

• The authors show late-life mating efficiency and maintained exploratory behavior in day 5 skn-1b males. It would be more informative to repeat the food-leaving assay at day 5 rather than exploration alone, as leaving more directly reports mate-searching prioritization.

• A locomotion control would help confirm that skn-1b effects are specific to mating motivation rather than general hyperactivity.

• For the odr-10 RNAi experiment (Fig 4G), odr-10 is already very low in males, so additivity would be hard to detect. It’s also unclear how to interpret the negative result given that WT vs skn-1b males do not differ in exploration to begin with. Running the assay under starvation, or using a spot-leaving paradigm, may reveal a clearer epistatic relationship. Clarifying the RNAi exposure timing would also help.

• Overall, stronger behavioral controls would help support the neuroendocrine model (skn-1b → daf-7 → odr-10), or alternatively the claims linking skn-1b to odr-10 could be softened.

B. Mitochondrial Biology

• The mitochondrial section shows interesting hermaphrodite-specific changes (structured networks, altered respiration) but is not well integrated with the neuronal skn-1b story.

• The authors do not discuss how a neuronal isoform controls muscle mitochondria, nor whether this impacts behavior or energetics.

• fzo-1 and drp-1 are DEGs in the male dataset, suggesting possible changes in fission/fusion, which could provide mechanistic insight if explored.

• Section feels descriptive rather than mechanistically linked.

C. Transcriptomic Analysis

• The dataset is rich but underutilized for mechanistic insight. The authors should discuss why a neuron-specific isoform would drive ~3,000 DEGs in males and only 44 in hermaphrodites.

• The authors note up-regulation of pkd-2 and lov-1 in skn-1b males in the discussion; both have established roles in mating. Highlighting these and other male-behavioral genes in their analysis would strengthen the connection to phenotype.

• Additional mining of tissue/neuronal expression databases would improve insight. For example, str-148 is downregulated in both sexes in skn-1b mutants and is AWA-specific, with reports of expression in male CP9 neurons, which could plausibly contribute to sensory/mating phenotypes.

• Baseline WT male vs WT hermaphrodite comparisons for skn-1b-dependent genes would help contextualize the male-biased DEG profile.

• Validation of a subset of DEGs (qPCR, reporter, behavioral experiments) would substantially strengthen confidence.

Minor Concerns / Edits

• Comment on other skn-1 isoforms and whether they were tested in males.

• It would be helpful to compare hermaphrodite vs male skn-1b expression levels in WT and mutants.

• Include hermaphrodite off-food exploration data or reference if previously published.

• Please correct for accuracy: hermaphrodite reproduction is limited by self-sperm depletion, not oocyte number.

• Venn diagrams should be drawn correctly (proportional circles and overlaps, not one size for all)

• Fig notes:

o S2B–C: images of male actin filaments would be informative.

o Fig 2E: add title/indicator that this assesses mitochondrial function.

o Clarify Fig 2F (herm) vs Fig 2J (male) in figure/legend.

o Fig 3A–B: consider matching PCA/venn color schemes. A bar plot may be clearer than a venn diagram.

o Fig 3C: could highlight male-behavior genes and/or show absence in herm dataset.

o Fig 3D–E: not cited?

o Several references to Fig 5 appear to refer to Fig 4.

o Fig 6 (model): changes to the directional arrows for male phenotypes (mate-searching ↑, mating success ↑) and herm phenotypes (exploration ↓) would perhaps make more sense.

• Methods need more detail on: genotypes sequenced, how males were generated, and genotype of worms used for mating assays.

• Colors are confusing- use separate colors rather than slightly different shades for more clarity.

**********

**Have all data underlying the figures and results presented in the manuscript been provided?**

Large-scale datasets should be made available via a public repository as described in the *PLOS Genetics*  data availability policy, and numerical data that underlies graphs or summary statistics should be provided in spreadsheet form as supporting information.

Reviewer #1: Yes

Reviewer #2: **No:**

**********

PLOS authors have the option to publish the peer review history of their article (what does this mean?). If published, this will include your full peer review and any attached files.

If you choose “no”, your identity will remain anonymous but your review may still be made public.

**Do you want your identity to be public for this peer review?**  For information about this choice, including consent withdrawal, please see our Privacy Policy.

Reviewer #1: No

Reviewer #2: No

[NOTE: If reviewer comments were submitted as an attachment file, they will be attached to this email and accessible via the submission site. Please log into your account, locate the manuscript record, and check for the action link "View Attachments". If this link does not appear, there are no attachment files.]

**Figure resubmission:**

**Reproducibility:**

To enhance the reproducibility of your results, we recommend that authors of applicable studies deposit laboratory protocols in protocols.io, where a protocol can be assigned its own identifier (DOI) such that it can be cited independently in the future. Additionally, PLOS ONE offers an option to publish peer-reviewed clinical study protocols. Read more information on sharing protocols at https://plos.org/protocols?utm_medium=editorial-email&utm_source=authorletters&utm_campaign=protocols

# Author comment: Author response to Decision Letter 1

**DOI:** 10.1371/journal.pgen.1012239.r002

11 Jun 2026

Attachment Submitted filename: PLoS Genetics REVIEW REBUTTAL.docx

# Peer review: Decision Letter 1

**DOI:** 10.1371/journal.pgen.1012239.r003

**Authors:** Monica P. Colaiácovo, Benjamin Podbilewicz

6 Jul 2026

Dear Dr Tullet,

We are pleased to inform you that your manuscript entitled "Neuronal Nrf/SKN-1B controls a sexually dimorphic neuroendocrine pathway for C. elegans exploratory behaviour." has been editorially accepted for publication in PLOS Genetics. Congratulations!

Before your submission can be formally accepted and sent to production you will need to complete our formatting changes, which you will receive in a follow up email. Please be aware that it may take several days for you to receive this email; during this time no action is required by you. Please note: the accept date on your published article will reflect the date of this provisional acceptance, but your manuscript will not be scheduled for publication until the required changes have been made.

Once your paper is formally accepted, an uncorrected proof of your manuscript will be published online ahead of the final version, unless you’ve already opted out via the online submission form. If, for any reason, you do not want an earlier version of your manuscript published online or are unsure if you have already indicated as such, please let the journal staff know immediately at plosgenetics@plos.org.

In the meantime, please log into Editorial Manager at https://www.editorialmanager.com/pgenetics/, click the "Update My Information" link at the top of the page, and update your user information to ensure an efficient production and billing process. Note that PLOS requires an ORCID iD for all corresponding authors. Therefore, please ensure that you have an ORCID iD and that it is validated in Editorial Manager. To do this, go to ‘Update my Information’ (in the upper left-hand corner of the main menu), and click on the Fetch/Validate link next to the ORCID field.  This will take you to the ORCID site and allow you to create a new iD or authenticate a pre-existing iD in Editorial Manager.

If you have a press-related query, or would like to know about making your underlying data available (as you will be aware, this is required for publication), please see the end of this email. If your institution or institutions have a press office, please notify them about your upcoming article at this point, to enable them to help maximise its impact. Inform journal staff as soon as possible if you are preparing a press release for your article and need a publication date.

Thank you again for supporting open-access publishing; we are looking forward to publishing your work in PLOS Genetics!

Yours sincerely,

Benjamin Podbilewicz

Academic Editor

PLOS Genetics

Monica Colaiácovo

Section Editor

PLOS Genetics

Aimée Dudley

Editor-in-Chief

PLOS Genetics

Anne Goriely

Editor-in-Chief

PLOS Genetics

www.plosgenetics.org

BlueSky: @plos.bsky.social

----------------------------------------------------

Comments from the reviewers (if applicable):

Reviewer's Responses to Questions

**Comments to the Authors:**

Reviewer #1: The authors have satisfactorily addressed my initial concerns and the manuscript is written in a much more clear manner now.

I only found a few typos that need fixing:

- In figure 1G the hermaphrodite symbol has been replaced by a square (some issue with the font i imagine).

-In the section "SKN-1B regulates male-specific neuroendocrine signalling to control

exploratory behaviour' the figure number referred to should be 4, not 5

- In figure 4 D, E and F it would help if there was a label stating in what neuron or what gene the expression of which is being measured

- In figure 5, the phenotype of basal O2 consumption in hermaphrodites should be down rather than up since skn-1b mutants have higher levels

Reviewer #2: The authors have addressed my concerns.

**********

**Have all data underlying the figures and results presented in the manuscript been provided?**

Large-scale datasets should be made available via a public repository as described in the *PLOS Genetics*  data availability policy, and numerical data that underlies graphs or summary statistics should be provided in spreadsheet form as supporting information.

Reviewer #1: Yes

Reviewer #2: Yes

**********

PLOS authors have the option to publish the peer review history of their article (what does this mean?). If published, this will include your full peer review and any attached files.

If you choose “no”, your identity will remain anonymous but your review may still be made public.

**Do you want your identity to be public for this peer review?**  For information about this choice, including consent withdrawal, please see our Privacy Policy.

Reviewer #1: No

Reviewer #2: No

----------------------------------------------------

**Data Deposition**

If you have submitted a Research Article or Front Matter that has associated data that are not suitable for deposition in a subject-specific public repository (such as GenBank or ArrayExpress), one way to make that data available is to deposit it in the Dryad Digital Repository. As you may recall, we ask all authors to agree to make data available; this is one way to achieve that. A full list of recommended repositories can be found on our website.

The following link will take you to the Dryad record for your article, so you won't have to re‐enter its bibliographic information, and can upload your files directly:

http://datadryad.org/submit?journalID=pgenetics&manu=PGENETICS-D-25-01362R1

More information about depositing data in Dryad is available at http://www.datadryad.org/depositing. If you experience any difficulties in submitting your data, please contact help@datadryad.org for support.

Additionally, please be aware that our data availability policy requires that all numerical data underlying display items are included with the submission, and you will need to provide this before we can formally accept your manuscript, if not already present.

----------------------------------------------------

**Press Queries**

If you or your institution will be preparing press materials for this manuscript, or if you need to know your paper's publication date for media purposes, please inform the journal staff as soon as possible so that your submission can be scheduled accordingly. Your manuscript will remain under a strict press embargo until the publication date and time. This means an early version of your manuscript will not be published ahead of your final version. PLOS Genetics may also choose to issue a press release for your article. If there's anything the journal should know or you'd like more information, please get in touch via plosgenetics@plos.org.

# Editor report: Acceptance letter

**DOI:** 10.1371/journal.pgen.1012239.r004

**Authors:** Monica P. Colaiácovo, Benjamin Podbilewicz

PGENETICS-D-25-01362R1

Neuronal Nrf/SKN-1B controls a sexually dimorphic neuroendocrine pathway for C. elegans exploratory behaviour.

Dear Dr Tullet,

We are pleased to inform you that your manuscript entitled "Neuronal Nrf/SKN-1B controls a sexually dimorphic neuroendocrine pathway for C. elegans exploratory behaviour." has been formally accepted for publication in PLOS Genetics! Your manuscript is now with our production department and you will be notified of the publication date in due course.

The corresponding author will soon be receiving a typeset proof for review, to ensure errors have not been introduced during production. Please review the PDF proof of your manuscript carefully, as this is the last chance to correct any errors. Please note that major changes, or those which affect the scientific understanding of the work, will likely cause delays to the publication date of your manuscript.

Soon after your final files are uploaded, unless you have opted out or your manuscript is a front-matter piece, the early version of your manuscript will be published online. The date of the early version will be your article's publication date. The final article will be published to the same URL, and all versions of the paper will be accessible to readers.

For Research Articles, you will receive an invoice from PLOS for your publication fee after your manuscript has reached the completed accept phase. If you receive an email requesting payment before acceptance or for any other service, this may be a phishing scheme. Learn how to identify phishing emails and protect your accounts at https://explore.plos.org/phishing.

Thank you again for supporting PLOS Genetics and open-access publishing. We are looking forward to publishing your work!

With kind regards,

Sharmila Kamatchi

PLOS Genetics

On behalf of:

The PLOS Genetics Team

Carlyle House, Carlyle Road, Cambridge CB4 3DN | United Kingdom

plosgenetics@plos.org | +44 (0) 1223-442823

plosgenetics.org | Twitter: @PLOSGenetics
